# A New Gaze Estimation Method Considering External Light

**DOI:** 10.3390/s150305935

**Published:** 2015-03-11

**Authors:** Jong Man Lee, Hyeon Chang Lee, Su Yeong Gwon, Dongwook Jung, Weiyuan Pan, Chul Woo Cho, Kang Ryoung Park, Hyun-Cheol Kim, Jihun Cha

**Affiliations:** 1 Division of Electronics and Electrical Engineering, Dongguk University, 26 Pil-dong 3-ga, Jung-gu, Seoul 100-715, Korea; E-Mails: jmlee1019@dongguk.edu (J.M.L.); leehc@dongguk.edu (H.C.L.); adeptkang@naver.com (S.Y.G.); jung4759@gmail.com (D.J.); westlaopan90@gmail.com (W.P.); cho4400@dongguk.edu (C.W.C.); 2 Electronics and Telecommunications Research Institute, 218 Gajeong-ro, Yuseong-gu, Daejeon 305-700, Korea; E-Mails: kimhc@etri.re.kr (H.-C.K); jihun@etri.re.kr (J.C.)

**Keywords:** gaze tracking system, corneal SR, external light, pupil movable area

## Abstract

Gaze tracking systems usually utilize near-infrared (NIR) lights and NIR cameras, and the performance of such systems is mainly affected by external light sources that include NIR components. This is ascribed to the production of additional (imposter) corneal specular reflection (SR) caused by the external light, which makes it difficult to discriminate between the correct SR as caused by the NIR illuminator of the gaze tracking system and the imposter SR. To overcome this problem, a new method is proposed for determining the correct SR in the presence of external light based on the relationship between the corneal SR and the pupil movable area with the relative position of the pupil and the corneal SR. The experimental results showed that the proposed method makes the gaze tracking system robust to the existence of external light.

## 1. Introduction

Gaze tracking systems generally relate the pupil center to the eye’s gaze [1,2,3,4]. Pupils are characterized by low brightness, black color, and circular shape, which facilitates their segmentation in images. However, the brightness and color of a pupil may vary under the influence of light, especially in visible images, whereas near-infrared (NIR) images are not influenced by changes in the light. For that reason, most gaze tracking systems use an NIR camera and light to reduce the influence of visible light [1,2,3,4,5,6]. In conventional gaze tracking systems, by using an additional NIR illuminator, specular reflection (SR) usually occurs on the eye. The occurrence of SR suggests a reference point according to which the center of the pupil is consistently adjusted depending on the user’s head movement. Most people experience involuntary head movements, which means that people move their head unwittingly. Most gaze tracking systems use the center of the pupil in the image of two-dimensional (2D) coordinates, which is captured by a fixed camera. Therefore, head movement reduces the accuracy of the tracking as it changes the 2D coordinates of the center of the pupil. However, the use of SR enables the center of the pupil to be stabilized in spite of head movement. The SR appears in the same relative position on the eye due to the fixed position of the NIR illuminator. Thus, gaze tracking systems usually uses SR to reference a point in an image [7,8,9,10,11].

The NIR image captured by the NIR camera and illuminator is robust to the influence of environmental visible light. However, it is susceptible to light including wavelength similar to that of the NIR illuminator, such as halogen lamp and sunlight illumination, which frequently exist in the real environment. As most gaze tracking research is conducted in environments with restricted light, the NIR illuminator of the gaze tracking system would establish a single SR. On the other hand, in the real environment, the appearance of several SRs is possible, because external light leads to additional SRs that are similar to the SR obtained using the NIR illuminator of the gaze tracking system. Ultimately, multiple SRs complicate the detection of genuine SR caused by the NIR illuminator of the gaze tracking system.

Previous researches are categorized into two kinds: multiple illuminator- and single illuminator-based methods. The former [2,3,4,5,7,9,10,11,12,13,14] has better performance in discriminating the genuine SR from the imposter ones caused by external lights than the latter [1,8,15,16]. That is because the rough positions of genuine SRs can be predicted in the image by using the information of the setup positions of the multiple NIR illuminators. That is, the former method distinguishes between genuine SRs and imposter ones based on the positional relation between the multiple SRs generated by the multiple NIR illuminators. In general, user calibration is less required for the multiple illuminator-based method than the single illuminator-based method because the part of information of the individual variations of eye can be acquired by using multiple illuminators. However, the use of multiple illuminators consumes more power than one illuminator, and the size of system increases, thereby reducing the applicability of the gaze tracking systems. Multiple corneal SRs are capable of contorting the shape of the pupil when they appear on the pupil boundary, which can prevent the accurate location of the pupil’s center. In addition, more SRs usually occur on glasses surface when using multiple illuminators, which produces more imposter SRs in the image.

To overcome these problems, single illuminator-based methods are proposed [1,8,15,16]. However, their performance discriminating the genuine SR from the imposter ones caused by the external lights are lower than those by multiple illuminator-based methods because the positional relation between the SRs cannot be used in the single illuminator-based method.

Therefore, we newly propose single illuminator-based method for determining the correct SR in the presence of external light. The research is novel in the following five ways compared to previous work. First, by using component labeling and size filtering, the erroneous regions of external light in the captured image were removed even in cases where the external light is positioned behind the user. Second, a systematic analysis of the effect of external light was conducted by varying the position of the external light in relation to the display. Third, mathematical solutions were provided that discrimination between the correct SR and the imposter SR was possible. This was done by verifying the relationship between the corneal SR and the pupil movable area with the relative position of the pupil and the corneal SR. Fourth, a new pupil detection method is proposed based on the histogram of the eye region which is processed by an average filter. Fifth, through the experiments in various situations including external light of four directions, sunlight of three directions, people wearing glasses, and people experiencing various head movements (rotation and translation), the effectiveness of the proposed solution is proved. Table 1 shows the summarized comparisons of previous works and proposed method.

**Table 1:** Comparison of previous works and proposed methods.

Category		Strength	Weakness
Multiple illuminator-based method [2,3,4,5,7,9,10,11,12,13,14]		- It has the better performance of discriminating the genuine SR from the imposter ones by external lights than the single illuminator-based method. - Less user calibration is required than the single illuminator-based method.	- Consuming more power than the single illuminator-based method, and the size of system increases. - Multiple corneal SRs can contort the shape of the pupil when they appear on the pupil boundary, which can prevent the accurate location of the pupil’s center. - More SRs usually occur on the glasses surface by using multiple illuminators.
Single illuminator-based method	Not considering the imposter SRs by the external light [1,8,15,16]	- Less consumption of power for the illuminator, and smaller size of system. - Lower possibility of the shape of pupil being consorted by corneal SR. - Less SR occur on the glasses surface.	- The performances of discriminating the genuine SR from the imposter ones caused by the external lights are lower than those by multiple illuminator-based methods because the positional relation between the multiple SRs cannot be used.
	Considering the imposter SRs by the external light (**Proposed method**)	- Less consumption of power for the illuminator, and smaller size of system. - Lower possibility of the shape of pupil being consorted by corneal SR. - Less SR occur on the glasses surface. - High performance of discriminating the genuine SR from the imposter ones caused by the external lights.	- The various scenarios of the relative positions of the illuminator of gaze detection system and external light should be considered for the implementation of algorithm.

The rest of this paper is organized as follows: in Section 2, we explain the proposed gaze detection method robust to external light. Section 3 presents the experimental results of the proposed method with discussions. Finally, our conclusions are presented in Section 4.

## 2. Proposed Method

**Figure 1:**
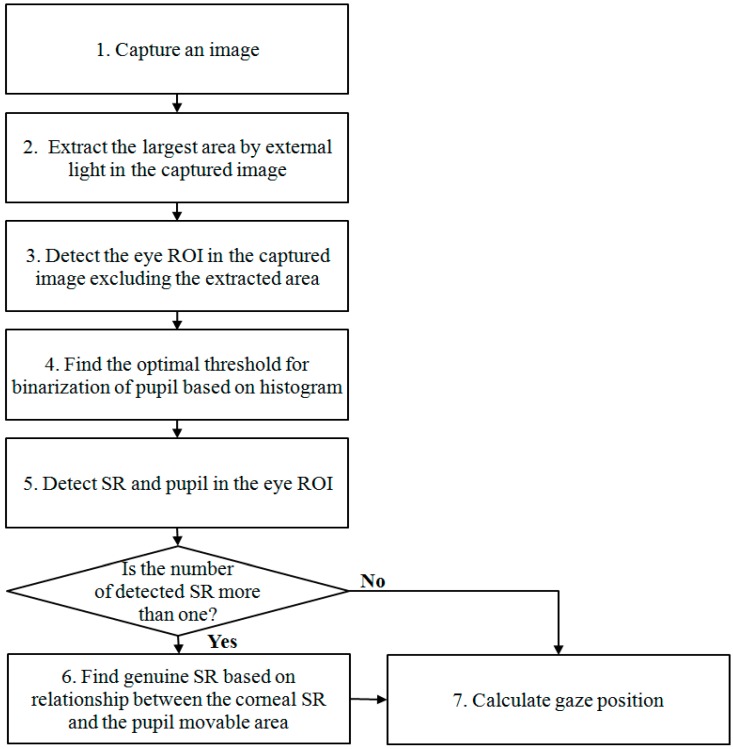
Flowchart of the proposed method.

Figure 1 depicts the overall procedure of the proposed method. With the captured image by the NIR illuminator and camera, the region by background external light in the captured image is detected (Step 2; see Section 2.2). Then, our system determines the eye region of interest (ROI) based on the candidate of SRs in the captured image where the region by background external light is excluded.

Because the pupil is usually darker than other regions such as iris, eyelid, and skin, binarization methods are widely used for segmenting the pupil. However, the presence of external light complicates the determination of a threshold value for binarization. The eyelid casts a shadow over the eye when the eyelid is exposed to external light and the appearance of the eyelashes darkens. This causes the pixel value of the shadow and that of the eyelashes to assume a pixel value similar to that of the pupil, which makes it difficult to discriminate the pupil from other regions only by the binarization method. Thus, a histogram of eye region is used, which is smoothed by a mask (Step 4). An approach for determining the threshold value is shown in Section 2.3.

In the next step, the pupil and all the SR candidates are detected (Step 5). In general, more than one SR appear in the eyes as a result of external light; therefore, possible scenarios based on different positioning of external light sources are defined in Section 2.4. In cases in which there is more than one SR, the genuine SR is decided based on the relationship between the corneal SR and the pupil movable area (Step 6). The method that is used to realize this decision is discussed in Section 2.5. Then, gaze tracking is performed using the genuine SR and the center of the pupil (Step 7) (see the Section 2.5).

### 2.1. Reduction of the Effect by External Light Using BPF

Accurate pupil detection is indispensable to implement a high accuracy gaze detection system. In general, the pupil is not distinguishable from the surrounding iris region under visible light, especially in case of dark eyes of Asians and Africans, *etc.* However, it is more distinctive from the iris with the NIR light of longer wavelength [17]. Therefore, a NIR light and camera are used in conventional gaze detection systems.

**Figure 2:**
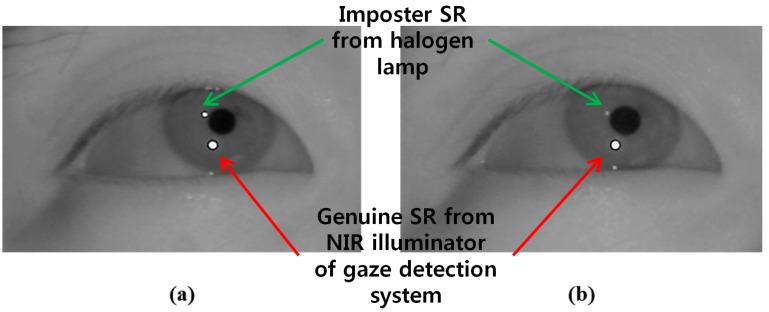
Eye region image under the illumination of halogen lamp (**a**) using LPF (**b**) using BPF.

**Figure 3:**
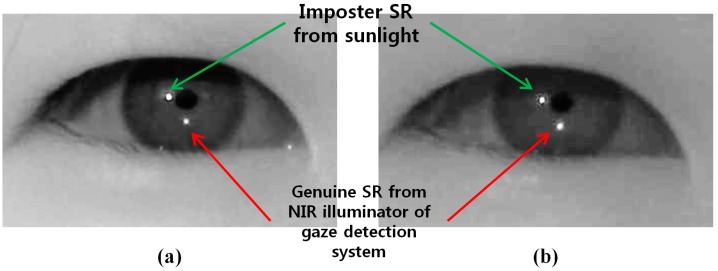
Eye region image under sunlight illumination (**a**) using LPF (**b**) using BPF.

In order to acquire an NIR image, most previous gaze tracking research use a long pass filter (LPF) which passes the NIR light (whose wavelength is longer than the visible light) into the camera. However, external lights such as halogen lamps or sunlight usually include a large amount of NIR component passing through the LPF, which can affect the performance of the gaze tracking system. To overcome this problem, we use a BPF for our gaze tracking camera (see the detailed specifications of BPF in the Section 3). As shown in Figure 2 and Figure 3, we can compare the images by the LPF with those by the BPF in case of the external light of halogen lamp and sunlight, respectively.

Figure 2a shows the captured image using LPF under the illumination of halogen lamp, in which the SR is created by passing the illuminations of halogen light through the LPF. This imposter SR appears similar to the genuine SR resulting from the NIR illuminator of the gaze detection camera. This indicates that it is difficult to determine which SR is the genuine one from the NIR illuminator. In general, the gaze tracking system calculates the gaze position using the disparity of pupil center based on the genuine SR position. Therefore, if the imposter SR is incorrectly selected as the genuine one, the error of gaze detection increases, and we can think that the case of Figure 2a can affect the performance of gaze tracking system.

On the other hand, the brightness of the SR created by passing the halogen illumination through the BPF is considerably reduced in the captured image (Figure 2b). Hence, the BPF is capable of reducing the effect of external light, because the wavelength it allows through corresponds to that of the NIR illuminator used in the gaze detection camera.

However, the system with the BPF has still limitations. Figure 3b shows the captured image in which BPF is used under sunlight illumination. The imposter SR resulting from passing sunlight through the BPF appears similar to the genuine SR resulting from the NIR illuminator of the gaze detection camera. That is because the sunlight includes a large amount of NIR component whose wavelength is similar to that of the NIR illuminator of the gaze detection system. Therefore, we propose the method of discriminating the imposter SR by the external light from the genuine one by the NIR illuminator of gaze detection system, as shown in Section 2.4 and Section 2.5.

### 2.2. The Method of Removing the Largest Area by Background External Light

**Figure 4:**
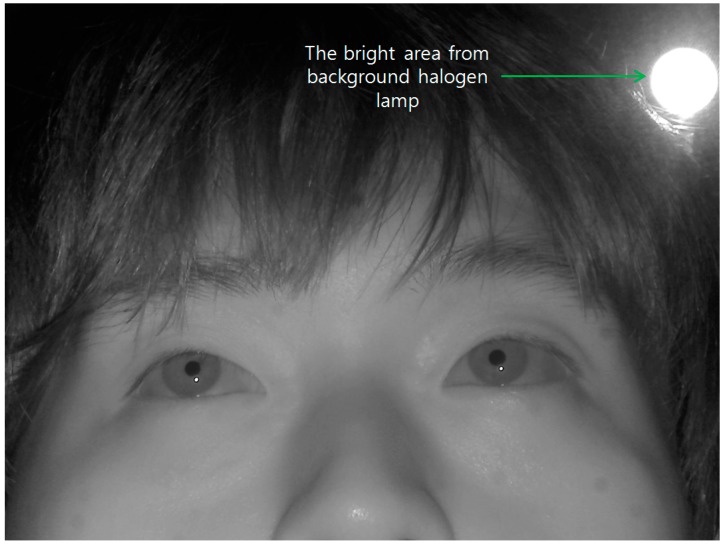
Captured image with halogen lamp located behind the user.

An external light source, such as a halogen lamp, which is located behind the user, can be displayed in the captured image as shown in Figure 4. Recently, halogen lamps have been widely used as interior illuminators, and a phenomena such as shown in Figure 4 frequently occur in real applications. In our gaze detection method, the eye ROI is firstly detected based on the candidate bright SRs in the captured image [18] because bright SRs caused by the NIR illuminator of the gaze detection system inevitably exist in eyes due to the high reflection rate of the cornea surface. Therefore, this kind of bright area caused by the background halogen lamp hinders the accurate detection of the bright SRs, and consequent the accuracy of gaze detection degrades.

**Figure 5:**
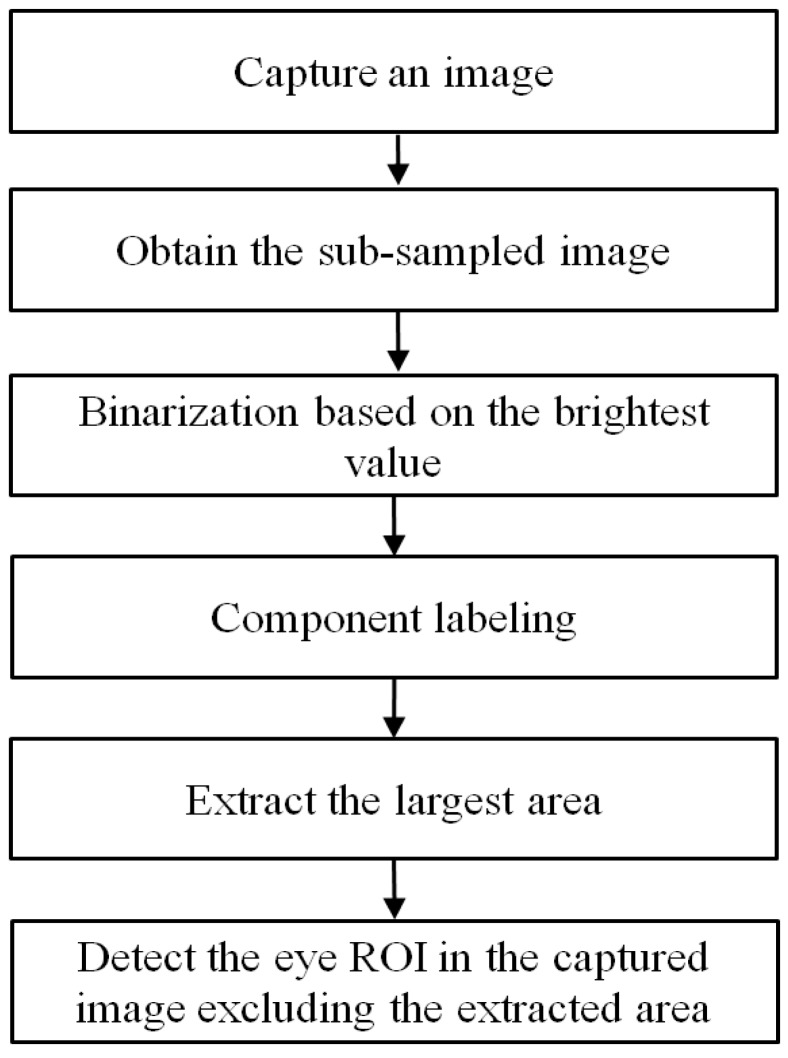
Flowchart to remove the area caused by external light behind user.

**Figure 6:**
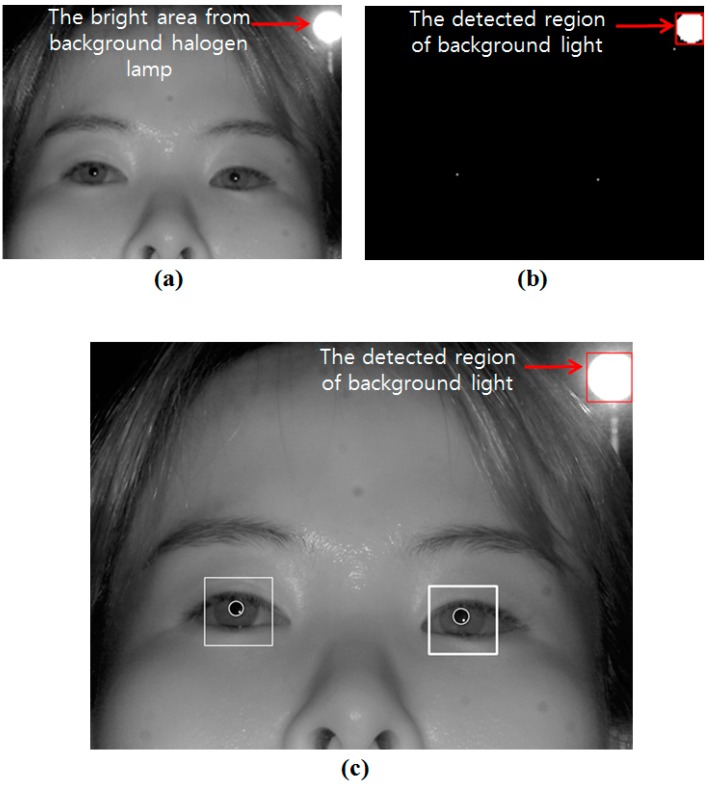
Step to remove external light behind user (**a**) the sub-sampled image of 1/8 size (**b**) binarized image (**c**) result image.

In order to solve this problem, we propose the method of removing the area caused by the external light as shown in Figure 5, which corresponds to Steps 2 and 3 in Figure 1. Considering that the area created by the background external light is usually bright and large as shown in Figure 4, the captured image is sub-sampled into one of 1/8 size in order to reduce the processing time. Then, the bright noises of small size on the facial skin disappear in the sub-sampled image, leaving the corneal SRs and the region illuminated by external light (Figure 6a). Because the size of the region created by the external light is much larger than the corneal SR, this region is readily located through binarization and component labeling (Figure 6b). Then, the eye ROIs are detected based on corneal SRs (Figure 6c) (Step 3 of Figure 1). The method for eye ROI detection is referred to previous research [18], and brief explanations are as follows.

To detect the eye ROI, the captured image (excluding the extracted area caused by background light) is segmented into M × N sub-blocks. Because the size of each sub-block is 32 × 30 pixels, and the size of captured image is 1600 × 1200 pixels, the values of M and N are 50 and 40, respectively. In each sub-block, the difference of maximum and minimum gray value is calculated, and all the sub-blocks are sorted in descending order based on the difference value. Then, the four sub-blocks of the highest 4th rank are determined as the candidate ones. Finally, two sub-blocks (whose average gray level is smaller than the average level of the four candidate sub-blocks) are determined as the eye sub-blocks. The position of the highest gray level in each eye sub-block is determined as the corneal SR positions, and based on these SR positions, the eye ROIs of 200 × 200 pixels are defined. In consecutive images, the detection of eye ROIs (based on corneal SR) is performed only in the pre-determined range considering the average Y position of the eye detected in previous frame, which can enhance the accuracy of the eye ROI detection [18].

### 2.3. Detecting the Center Positions of Pupil and Corneal SR

**Figure 7:**
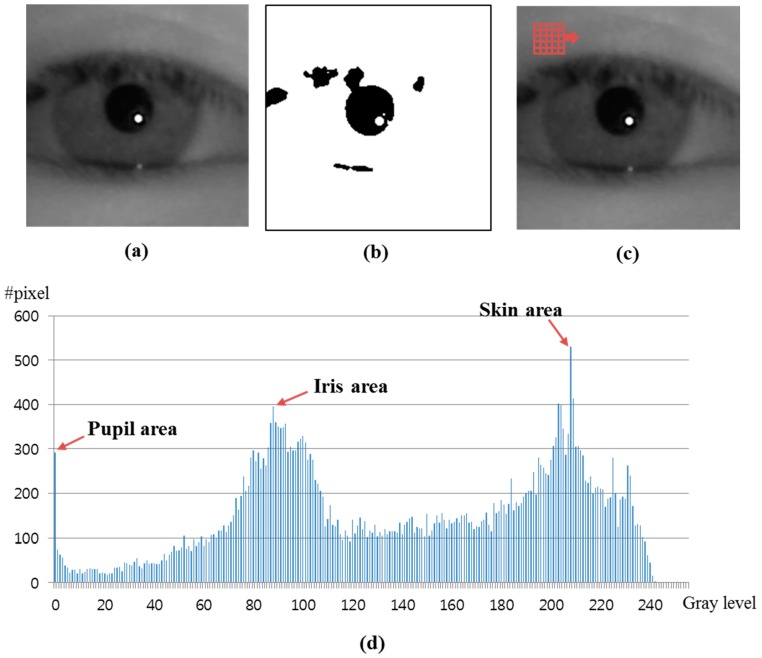
Binarized image using a static threshold and the eye region using the average filter. (**a**) original eye region; (**b**) binarized image using a static threshold; (**c**) result image obtained by the average filter; (**d**) histogram of (c).

In the detected eye candidate region, the pupil center is located as shown in Steps 4 and 5 of Figure 1. Accurate detection of the pupil center is very important in high accuracy gaze tracking systems. Many gaze tracking systems use a binarization method to locate the pupil center, which implies that the selection as to the optimal threshold value for binarization is the most important procedure. However, it is difficult, especially, in case of capturing an image with external light, because the eyelid can cast a shadow on the eye and the eyelashes appear darkened. The pixel values of the shadow and dark eyelashes are similar to that of the pupil, so if the gaze tracking system uses a static threshold for pupil binarization, areas other than the pupil can be included (Figure 7b).

We propose a new method for solving this problem by using a histogram of the eye region. We firstly apply an average filter of 5 × 5 pixels to the eye region as shown in Figure 7c. In general, because the size of shadows or eyelashes is smaller than the pupil area, areas other than the shadow or eyelash can be included in the filter of 5 × 5 pixels. Therefore, the effect of the shadow or eyelash is much reduced compared to the pupil by the average filter of 5 × 5 pixels, which can increase the discrimination between the pupil and any shadows or eyelashes.

**Figure 8:**
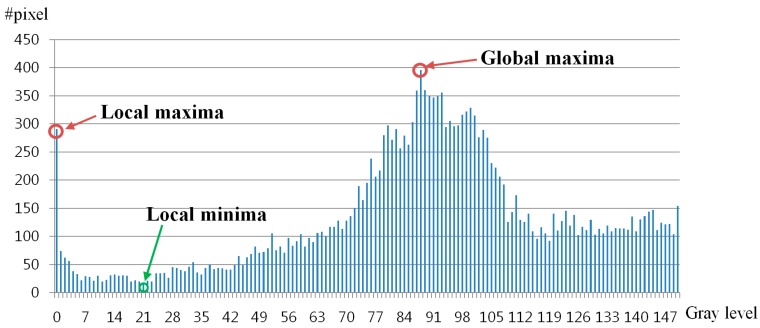
Detecting the position of local minima between the positions of global and local maxima.

Figure 7d shows the histogram of the Figure 7c. As shown in Figure 7d, the pixels with the highest number are the skin area because the largest area is skin in the eye ROI, as shown in Figure 7c. In addition, the pixels (with the second highest number) whose gray level is usually lower than the skin are the iris area. The pixels (with the third highest number) whose gray level is lower than the iris and skin are the pupil area. Because the absorption rate of NIR light in the iris is higher than that in the skin and sclera, the gray level of the iris is usually lower than the skin and sclera, as shown in Figure 7a [17]. Based on this, our system finds the positions of the global and local maxima within the range whose gray level is less than the skin. For example, as shown in Figure 7d, because the gray level of skin area is about 207, our system finds the positions of the global and local maxima in the range of gray level from 0 to 192 considering the gray level difference (greater than 15 (207–192)) between the skin and iris. Then, the global and local maxima correspond to the iris and pupil areas, respectively, as shown in Figure 7d and Figure 8. For example, in Figure 7 and Figure 8, the global and local maxima are obtained as about 89 and 1, respectively.

**Figure 9:**
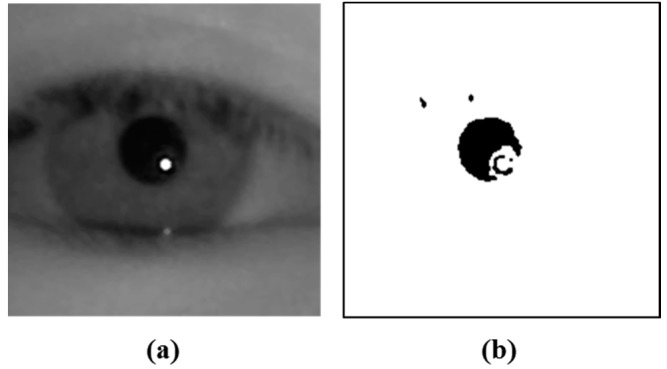
Binarized image using the local minima value as threshold.

Between the positions of the global and local maxima, the position of local minima (whose number of pixels is the smallest) can be detected, which represents the boundary between the iris and pupil areas. Therefore, our system set this position of local minima as the threshold for binarization. Figure 9b shows the binarized image using this local minima value as threshold. By comparing Figure 7b and Figure 9b, we can find that our binarized method can make the pupil area more separable from other areas than the method using a static threshold.

**Figure 10:**
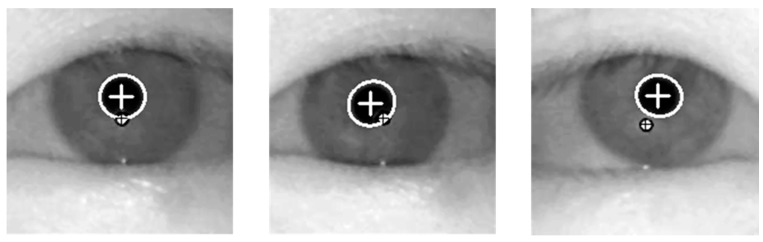
The examples of detected pupil center and corneal SRs.

Using the binarized image of Figure 9b, the pupil center is obtained through further procedures including morphological operation, component labeling, canny edge detection, and ellipse fitting [18]. In addition, with the detected pupil center, the search area for corneal SR is defined, and corneal SR is located within this area by binarization and component labeling [18]. Figure 10 shows the examples of detected pupil center and corneal SRs.

### 2.4. The Possible Cases of Positions of External Light

As shown in the Figure 3, external light produces imposter SRs in the eye image, which makes it difficult for our system to discriminate the genuine corneal SR from the imposter ones. To overcome this problem, we define the possible external light position cases as follows.

**Figure 11:**
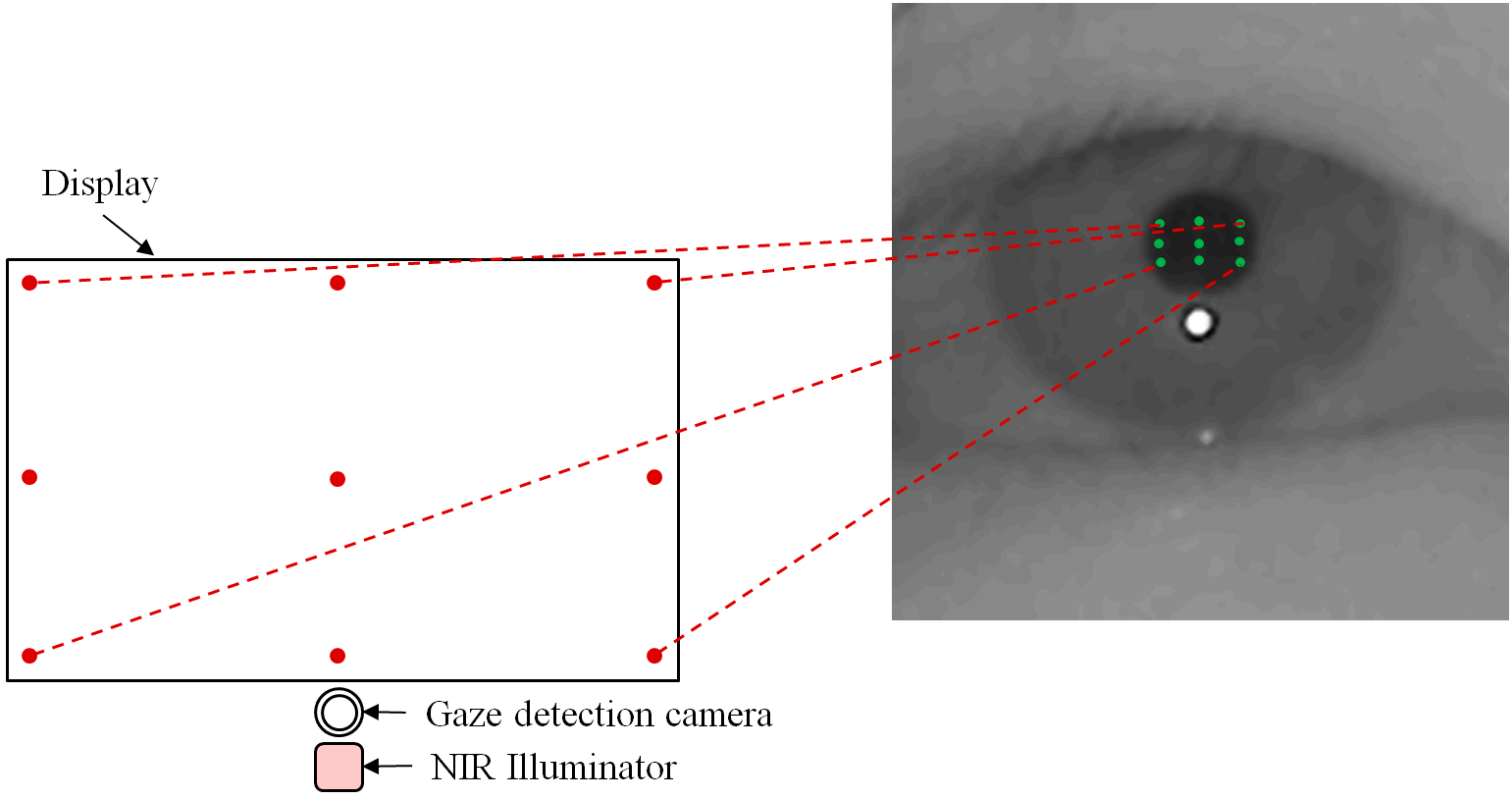
Nine pupil centers which are obtained when a user gazes at nine calibration points.

Most gaze tracking systems require a user to undergo an initial calibration process, which makes the user look at several (six or nine) pre-determined points on a monitor screen. Through this process, the relationship between the pupil movable area and monitor screen is acquired. Our system requires each user to gaze at nine positions as the initial calibration process. In the right image of Figure 11, the nine green points on the pupil represent the nine pupil centers which are obtained when a user gazes at nine calibration points on the display as shown in the left image of Figure 11.

**Figure 12:**
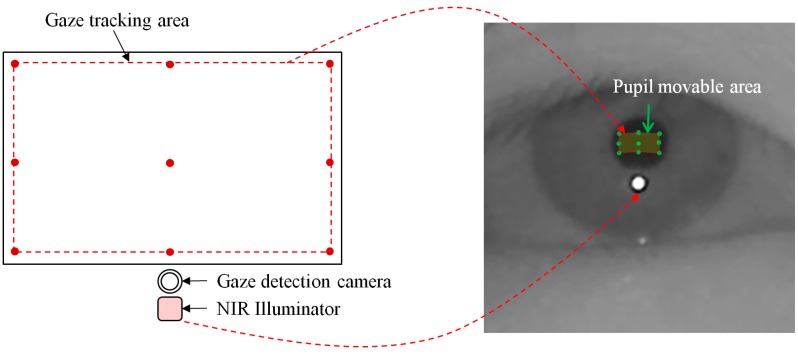
The possible area of gaze tracking.

**Figure 13:**
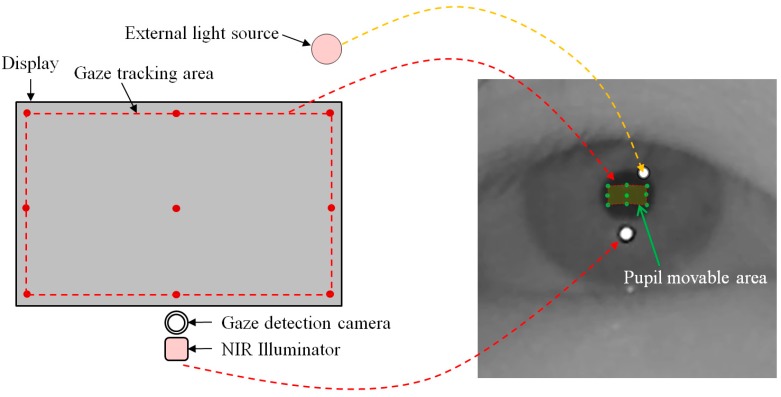
Eye image in presence of external light when using a monitor display.

Based on the nine green points of the pupil center (in the right image of Figure 11), we can define the pupil movable area (in the right image of Figure 12) which corresponds to the gaze tracking area of the left image of Figure 12. Because all the calibration point positions are close to the boundary of the monitor display, as shown in Figure 12, the pupil center exists within the pupil movable area in the most cases when a user gazes at any position on the monitor display. Based on this, we can consider the case that the external light source exists outside the monitor display as shown in Figure 13.

With the external light outside the monitor display, the SR (on the eye) that is created by external light appears as shown in Figure 13. However, if the external light source is positioned behind the monitor display, it does not appear in the eye image, because the monitor display hides the external light source. That is, if we use the gaze tracking system with a monitor display, external light cannot exist inside the gaze tracking area on the monitor display and the consequent SR caused by the external light cannot occur in the corresponding pupil movable area in the eye image as shown in Figure 13. Based on this characteristic, the detailed explanations of how to discriminate the imposter SR caused by the external light from the genuine one by the NIR illuminator of the gaze tracking system in the case a monitor display is used are included in the next section.

### 2.5. Discriminating the Genuine SR from the Imposter One, and Calculating Gaze Position

**Figure 14:**
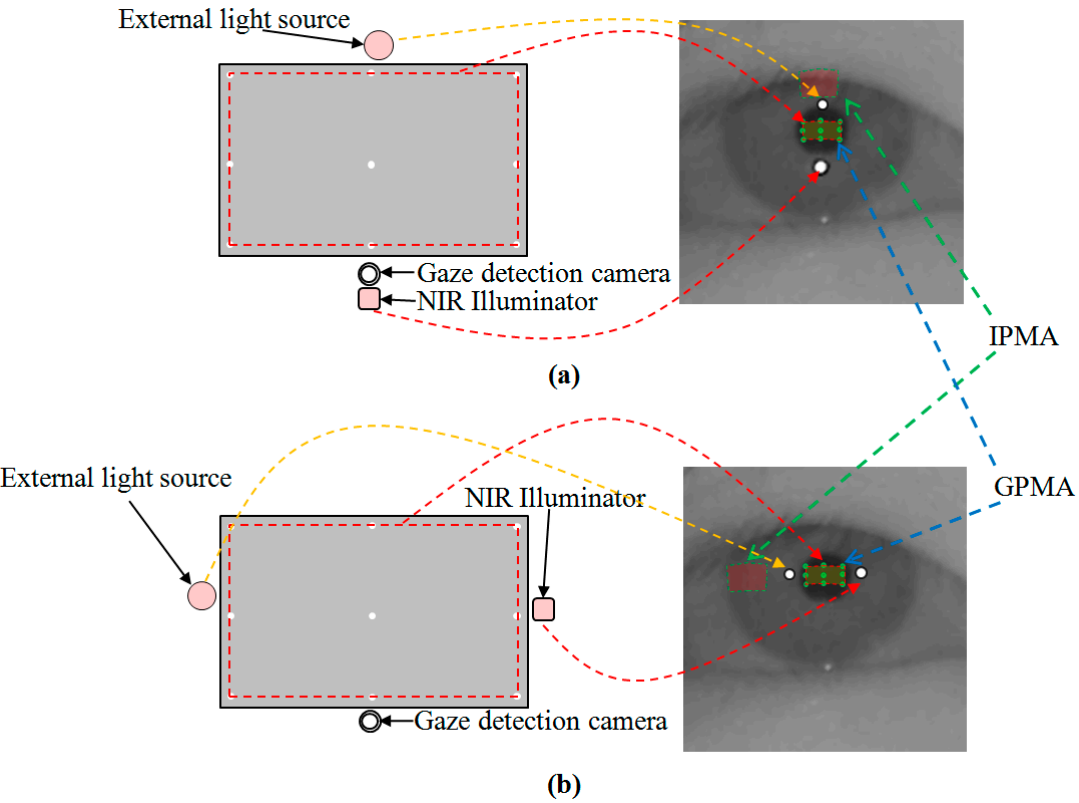
First scenario (external light source exists at the opposite position to the NIR illuminator based on the monitor display), and corresponding GPMA and IPMA: (**a**) External light source exists in the upper position of monitor display (**b**) External light source exists in the left position of monitor display.

In this section, we explain the proposed method to discriminate the genuine SR from the imposter ones caused by the external light in the case a monitor display is used as shown in Figure 13. Because we usually know the set-up position of the NIR illuminator of the gaze tracking system based on the monitor screen, we can assume two pupil movable areas based on the SR. One of the areas is based on the genuine SR created by the NIR illuminator of the gaze tracking system. The other is based on the imposter SR created by external light. We call the former and latter a genuine pupil movable area (GPMA) and an imposter pupil movable area (IPMA), respectively, for convenience. Examples of the GPMA and IPMA are shown in Figure 14. In Figure 14a, because we know that the set-up position of the NIR illuminator is below the monitor screen, we can define two pupil movable areas above two SRs as shown in the right image of Figure 14a. In Figure 14b, because we know that the set-up position of the NIR illuminator is to the right of the monitor screen, we can define two pupil movable areas left as two SRs as shown in the right image of Figure 14b. Like this method, we can consider four scenarios. In the first scenario, the external light source and NIR illuminator are at opposite positions with respect to the monitor screen, as shown in Figure 14. Figure 14a is the case which occurs most frequently in real applications of gaze tracking systems with external light.

As explained before, because all the calibration positions are close to the boundary of the monitor display, as shown in Figure 14, the pupil center exists within the GPMA when a user gazes at any position on the monitor display. In addition, the IPMA does not overlap with the GPMA in the case of the first scenario. Therefore, we can determine the pupil movable area (where the pupil center belongs) as the GPMA, and the corresponding SR (which is related to the GPMA) can be determined as the genuine SR created by the NIR illuminator of the gaze tracking system. For example, in Figure 14a, the pupil center inevitably belongs to the GPMA, and we know that the set-up position of the NIR illuminator is below the monitor screen (the genuine SR by the NIR illuminator is positioned below the GPMA in the eye image because the monitor screen corresponds to the GPMA). Therefore, the SR of the lower position in the eye image can be determined as the genuine one by the NIR illuminator. This method is expressed by Equation (1):

If ((PMA_1_ ∩ PMA_2_ = Ø) and (P(x, y) ∈ PMA_1_)), then PMA_1_ is GPMA (1)

where P(x, y) is the center of pupil. If there is no common area between PMA_1_ and PMA_2_, and P(x, y) is within the PMA_1_, then PMA_1_ can be determined as GPMA. In Equation (1), we designate the two pupil movable areas as PMA_1_ and PMA_2_, respectively.

**Figure 15:**
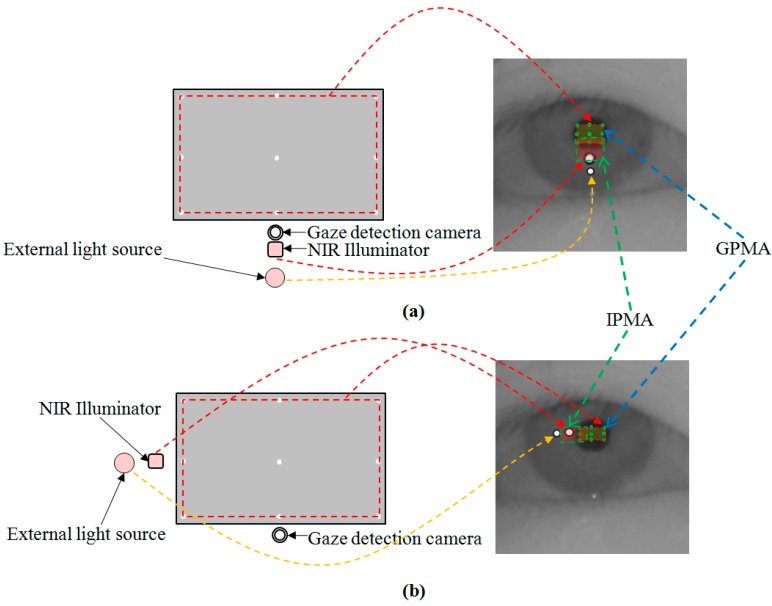
Second scenario (external light source exists at the same direction as the NIR illuminator based on the monitor display), and corresponding GPMA and IPMA: (**a**) External light source exists in the lower position of monitor display; (**b**) External light source exists in the left position of monitor display.

Figure 15 shows the second scenario. In this case, the external light source and the NIR illuminator are positioned in the same direction with respect to the monitor display. Because the external light source and the NIR illuminator are positioned in the same direction, and their positions are close each other, the GPMA and IPMA have an overlapped area, as shown in Figure 15. The method to discriminate the genuine SR is then given by Equation (2):

If ((PMA_1_ ∩ PMA_2_ ≠ Ø) and (R(x, y) ∈ PMA_2_) and (P(x, y) ∈ PMA_1_)), then PMA_1_ is GPMA (2)

where P(x, y) and R(x, y) are the centers of pupil and SR, respectively. 

If there is common area between PMA_1_ and PMA_2_, R(x, y) is within the PMA_2_, and P(x, y) is within the PMA_1_, then PMA_1_ can be determined as GPMA. From that, we can determine the corresponding SR (which is related to the GPMA) as the genuine SR produced by the NIR illuminator of the gaze tracking system.

**Figure 16:**
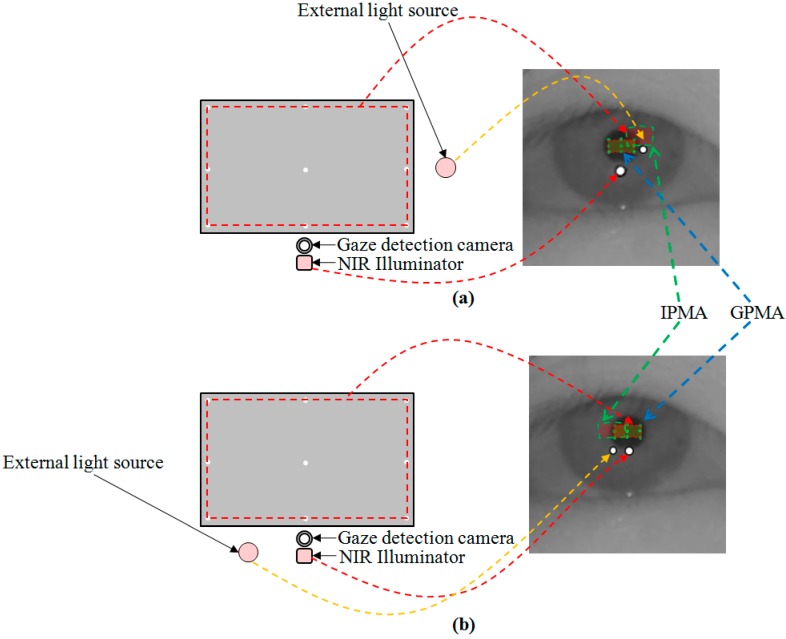
Third scenario (external light source exists in the right or left position of the NIR illuminator), and corresponding GPMA and IPMA: (**a**) External light source exists in the right position of the NIR illuminator; (**b**) External light source exists in the left position of the NIR illuminator.

In the third scenario, the GPMA and IPMA have some common area as shown in the right image of Figure 16. Therefore, if the pupil center belongs to the common area, it is difficult to determine which one is GPMA among two pupil movable areas, which can cause gaze detection errors. However, because the size of the common area is smaller compared to the GPMA, this case does not frequently occur. In other cases, because the pupil centers belong to only the GPMA, we can easily determine the GPMA and corresponding correct SR.

**Figure 17:**
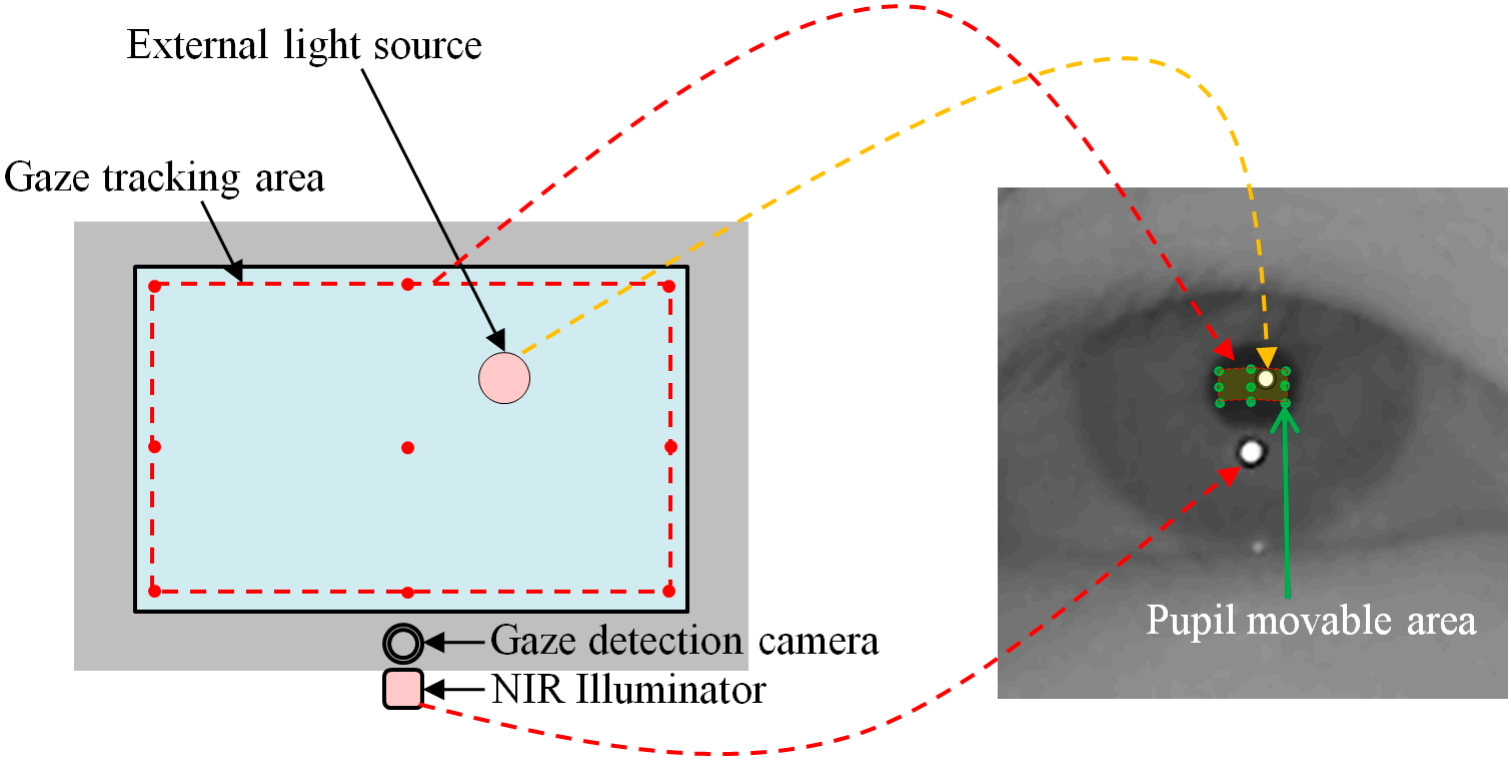
Eye image in presence of external light in a car environment.

As the fourth scenario, considering the gaze tracking system in a car environment, the gaze tracking area is not a monitor display but the frontal window as shown in Figure 17. In this case, the frontal window corresponds to the gaze tracking area, and only the external light source inside the frontal window can be seen on the eye image. Therefore, the external light source can exist only inside the gaze tracking area, and the consequent SR by the external light can occur only in the corresponding pupil movable area of the eye image as shown in Figure 17. Therefore, we can think the SR inside the pupil movable area as the imposter one produced by the external light, and easily discriminate this from the genuine SR created by the NIR illuminator of the gaze tracking system because the genuine SR cannot exist inside the pupil movable area, as shown in Figure 17.

**Figure 18:**
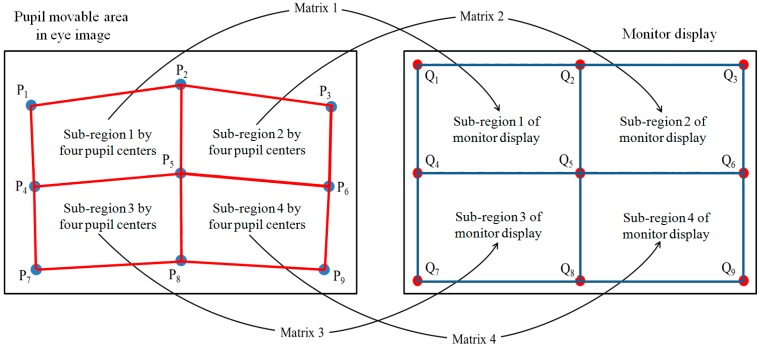
Relationship between four sub-regions defined by nine pupil centers and the corresponding four sub-regions of monitor display.

After obtaining the position of the genuine SR with the pupil center, the gaze position is calculated by the multiple geometric transform method [18]. As explained before, we obtain the nine positions of the pupil centers from the initial user calibration (during which each user gazes at the nine points on the monitor display), and the four relationships between each sub-region by the pupil centers and monitor sub-region are defined as shown in Figure 18. For example, the sub-region 2 by four pupil centers corresponds to monitor sub-region 2. This relationship is obtained by geometric transform, and four matrices of geometric transforms are acquired as shown in Figure 18 [3,18].

When calculating the gaze position, if the extracted pupil center is within the sub-region 1 according to the four pupil centers (the rectangle of P_1_P_2_P_5_P_4_ of Figure 18) our system calculates the gaze position on the monitor display by using geometric transform matrix 1. If it is within the sub-region 3 defined by the four pupil centers (the rectangle of P_4_P_5_P_8_P_7_ of Figure 18), our system calculates the gaze position by using geometric transform matrix 3 [18].

## 3. Experimental Results

**Figure 19:**
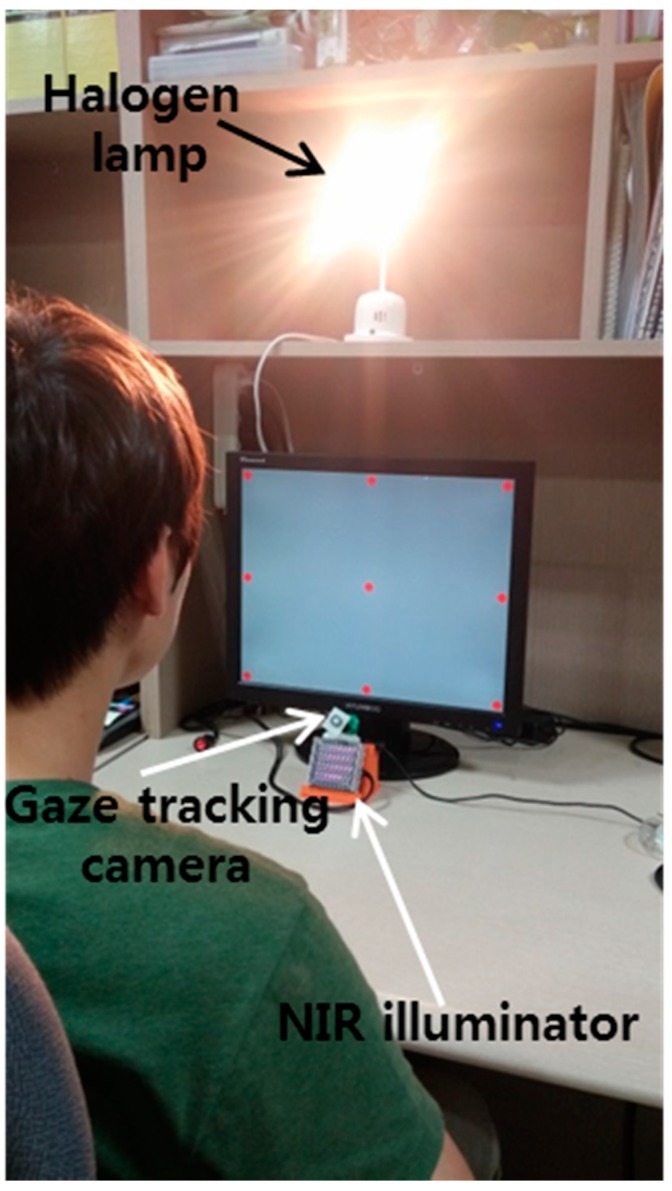
Experimental setup for measuring gaze tracking accuracy under a halogen lamp.

**Figure 20:**
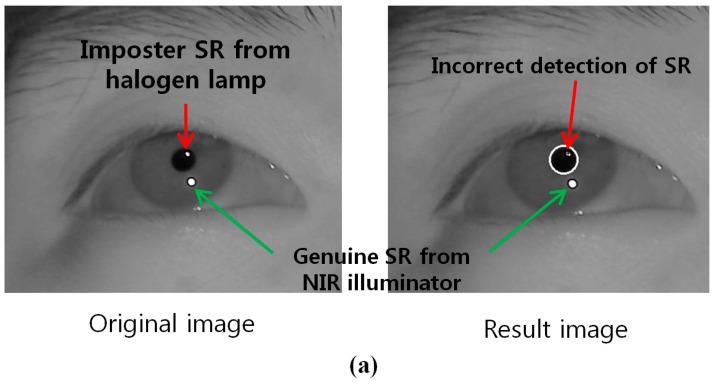

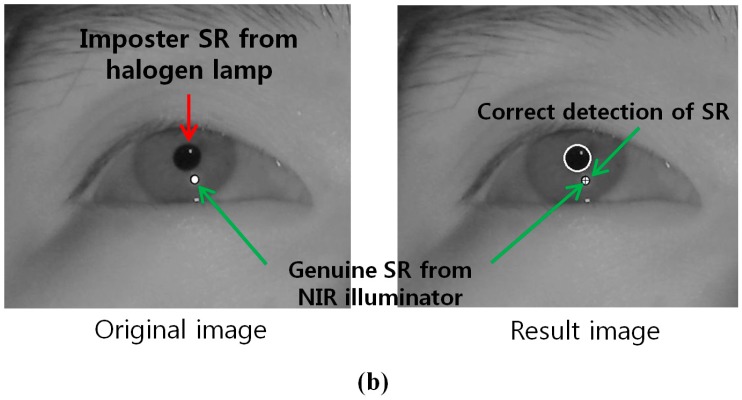
Resulting images which include the detected pupil and SR under a halogen lamp (**a**) using LPF (**b**) using BPF.

The gaze tracking system was evaluated on a desktop computer with an Intel core 2 quad Q8200 CPU of 2.33 GHz and 4 GB RAM. The images for the experiments were captured using our gaze tracking camera (C600 web-camera, Logitech, Newark, CA, USA) and the image size was 1600 × 1200 pixels [19]. Two types of filter such as LPF (Wratten Filter No. 89B, Kodak, Rochester, NY, USA) which passes the NIR light whose wavelength is the same as (longer than) 700 nm and BPF (passing range of 850 ± 25 nm) [20,21], and two types of halogen lamp [22,23] were used for the experiments. Because it is very difficult to obtain the images with sunlight according to the various directions of sunlight, we used high-powered halogen lamps for the experiments. Figure 19 shows our experimental setup for measuring the gaze tracking accuracy under a halogen lamp. The images while 10 people underwent three trials were collected for experiments, and the Z distance between the people and the monitor was about 50~90 cm. A 19-inch monitor with 1280 × 1024 pixels resolution was used for the experiments. Because most gaze tracking systems are used with a desktop (or laptop) computer indoors, we performed our experiments in this environment. As shown in Figure 20b, we can find that the effect of the imposter SR caused by the halogen lamp can be reduced by using the BPF on the camera, and the genuine SR created by the NIR illuminator can be correctly detected comparing to Figure 20a.

**Figure 21:**
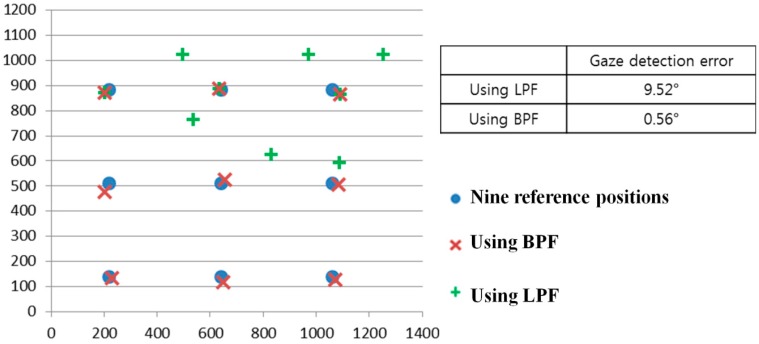
Comparisons of gaze detection errors by LPF and BPF.

As the first experiment, we compared the gaze detection error obtained by LPF with that obtained by BPF as shown in Figure 21. We measured the gaze detection error when each person gazes at nine reference points of Figure 21. In our research, the gaze detection error is calculated by the difference between each reference point and the calculated one by our gaze detection method. In the left image of Figure 21, we showed the reference points with the calculated ones by our gaze detection method using BPF and LPF, respectively. In this case, each calculated gaze position is obtained by averaging all the gaze positions calculated from 10 people doing three trials.

The experimental results showed that the gaze detection error using LPF is about 9.52°, which is much larger than that obtained using BPF (about 0.56°). As shown in Figure 21, we can confirm that the proposed gaze detection system with BPF outperforms that with LPF.

**Figure 22:**
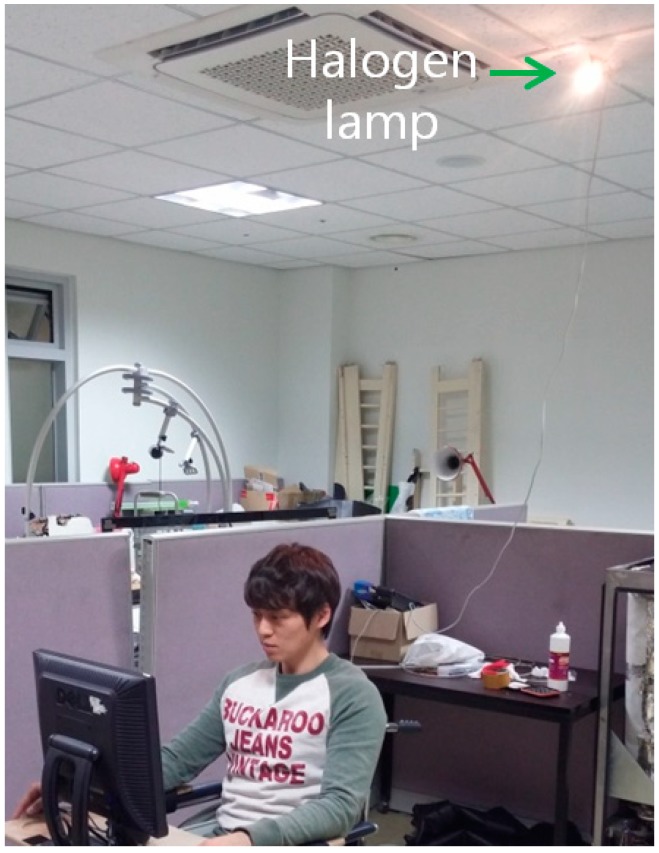
Experiments when a halogen lamp is located behind the user.

**Figure 23:**
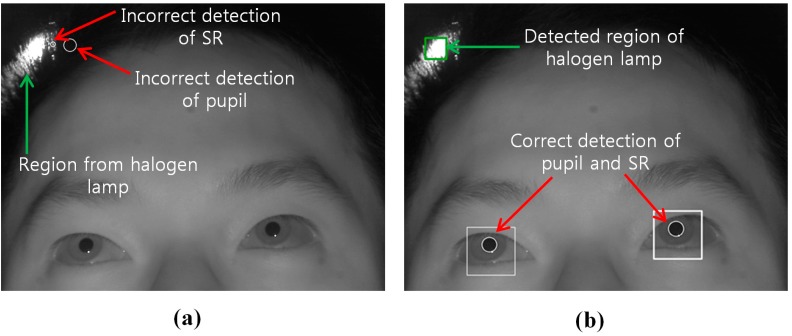
Result image of pupil and SR detection (**a**) not using our method; (**b**) using our method.

As the second experiment, we measured the gaze detection accuracy with or without the proposed method (removing the region of background external light as shown in Section 2.2) when a halogen lamp is located behind the user as shown in Figure 22. Ten persons performed the test with six trials (three trials when the background external light was positioned on the right based on the person, and the other three trials when it was positioned on the left based on the person). As shown in Figure 23, we can find that the correct pupil and SR can be detected with our method whereas the incorrect pupil and SR are detected without our method. Because some bright noises can exist around the detected region of background light as shown in Figure 23b, we increase the region with some margin, and detect the pupil and SR excluding this region.

**Figure 24:**
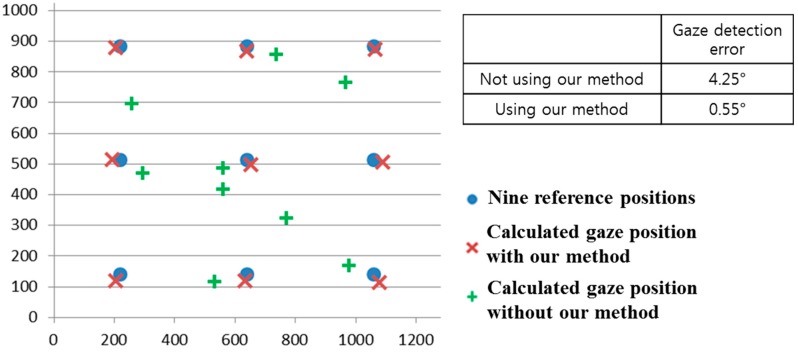
Comparisons of gaze detection errors without and with our method of removing the region of background external light as shown in Section 2.2.

We compared the gaze detection error with and without our method of removing the background external light region as shown in Figure 24. We measured the gaze detection error when each person gazes at the nine reference points in Figure 24. In the left image of Figure 24, we show the reference points, and the calculated ones using and not using our method. In this case, each calculated gaze position is obtained by averaging all the gaze positions calculated from 10 people undergoing three trials. The experimental results showed that the gaze detection error not using our method is about 4.25°, which is much larger than that (about 0.55°) using our method. As shown in Figure 24, we can confirm that the gaze detection system with our method outperforms that without our method.

**Figure 25:**
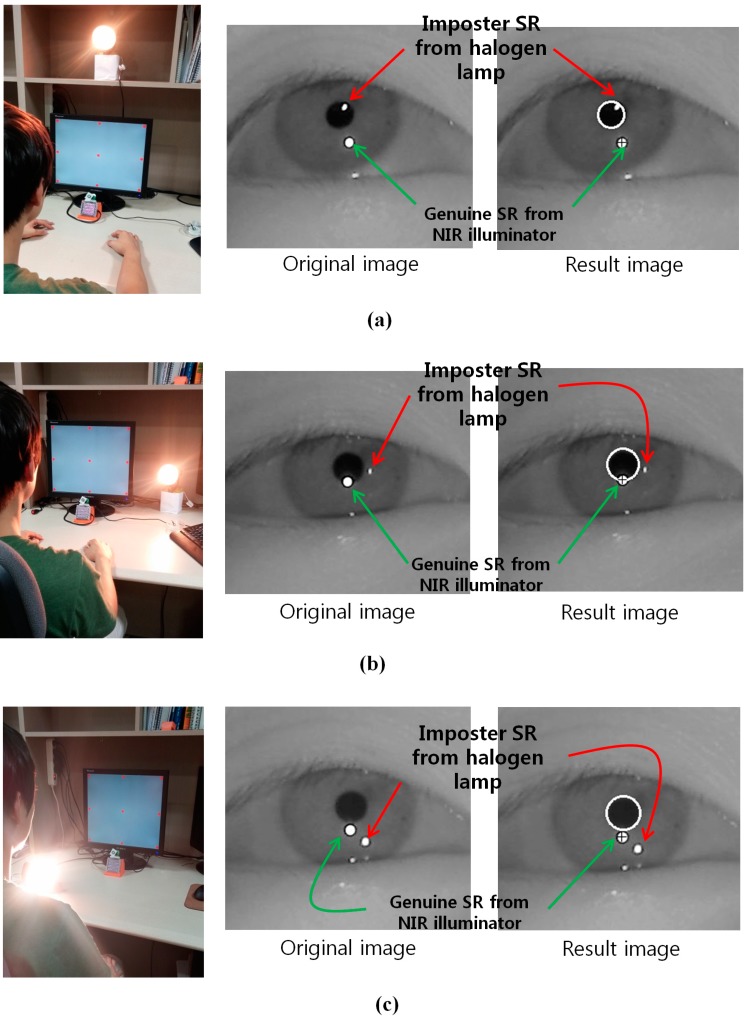

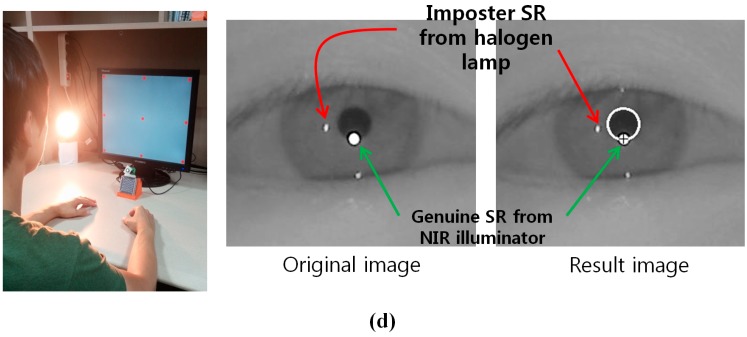
Result images where the pupil and SR are correctly detected in the presence of the halogen lamp at various positions (**a**) above monitor display (**b**) right of monitor display (**c**) below monitor display (**d**) left of monitor display.

As the next experiment, we compared the gaze detection accuracies with and without our method as described in Section 2.5. For that, we put the halogen lamp at various positions as shown in Figure 25. As shown in the resulting images (Figure 25a–d), we can find that genuine SR can be correctly detected by our method.

**Figure 26:**
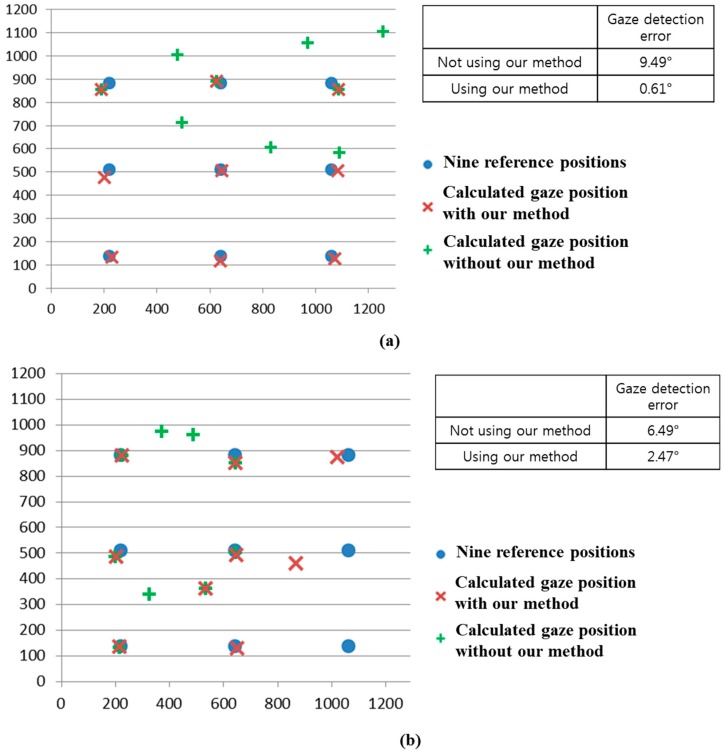

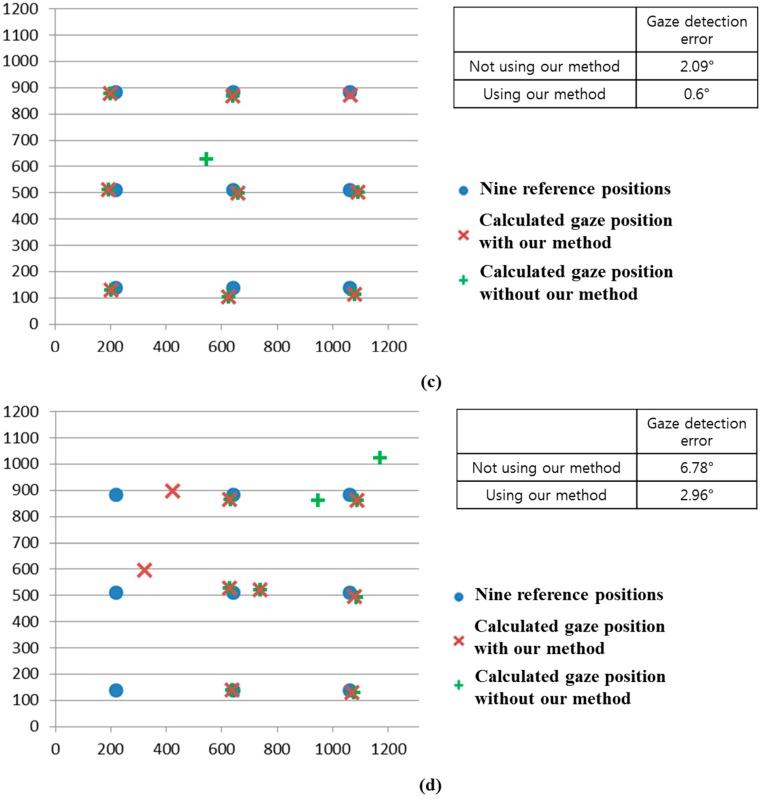
Comparisons of gaze detection errors without and with our method of Section 2.5 in case of the halogen lamp (**a**) above monitor display; (**b**) right of monitor display; (**c**) below monitor display; (**d**) left of monitor display.

We compared the gaze detection error with and without our method of Section 2.5 as shown in Figure 26. Figure 26a–d shows the gaze detection errors in the cases where the halogen lamp is placed at various positions. Like previous experiments, we measured the gaze detection error when each person gazes at the nine reference points of Figure 26. In the left images of Figure 26a–d, we show the reference points and the calculated ones using and not using our method. In these cases, each calculated gaze position is obtained by averaging all the gaze positions calculated from 10 people with three trials. Experimental results showed that the gaze detection errors using our method are much lower than those not using our method, irrespective of the positions of the halogen lamp. Although the gaze detection error with our method is much lower than that without our method, the errors with our method in case of Figure 26b,d are higher than those of Figure 26a,c. That is because these cases correspond to the third scenario of Figure 16, and the errors occur when the pupil center belongs to the common area of GPMA and IPMA.

As shown in Figure 16a, the common area has the characteristic that it exists in the right part of GPMA when the external light is positioned in the right based on the NIR illuminator. In addition, as shown in Figure 16b, the common area has the characteristic of being in the left part of GPMA when the external light is positioned in the left based on the NIR illuminator. Because the halogen lamp was positioned in the right based on the NIR illuminator in case of Figure 25b, this corresponds to the case of Figure 16a, and the resulting common area exists in the right part of GPMA. From this, we can know that the errors occur when the user gazes at the right reference positions (in this case, the probability of pupil center belonging to the common area increases because the common area is in the right part of GPMA) as shown in Figure 26b.

Like these, because the halogen lamp was positioned in the left based on the NIR illuminator in case of Figure 25d, this corresponds to the case of Figure 16b, and the resulting common area exists in the left part of GPMA. From this, we can know that the errors occur when the user gazes at the left reference positions (in this case, the probability of pupil center belonging to the common area increases because the common area is in the left part of GPMA) as shown in Figure 26d. In conclusion, we can confirm from the above experimental results that our gaze detection system is robust to the external light placement at various positions.

**Figure 27:**
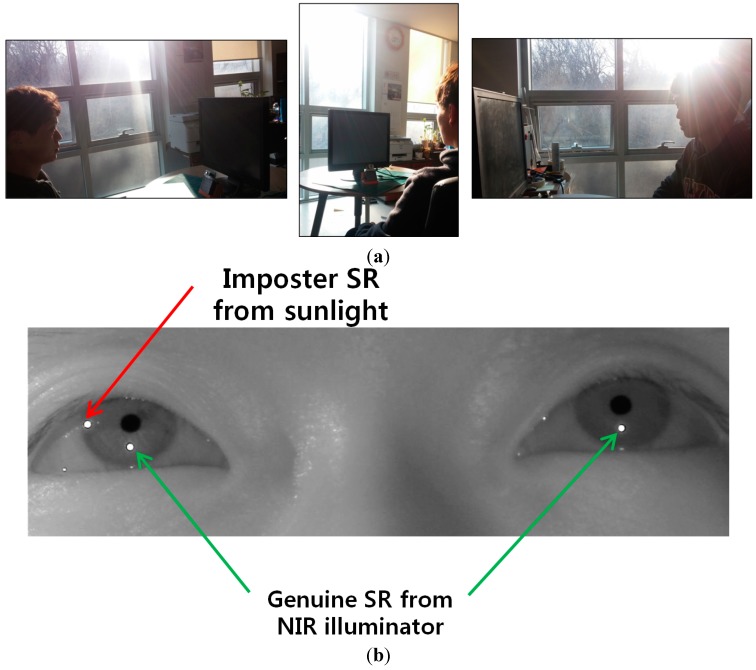

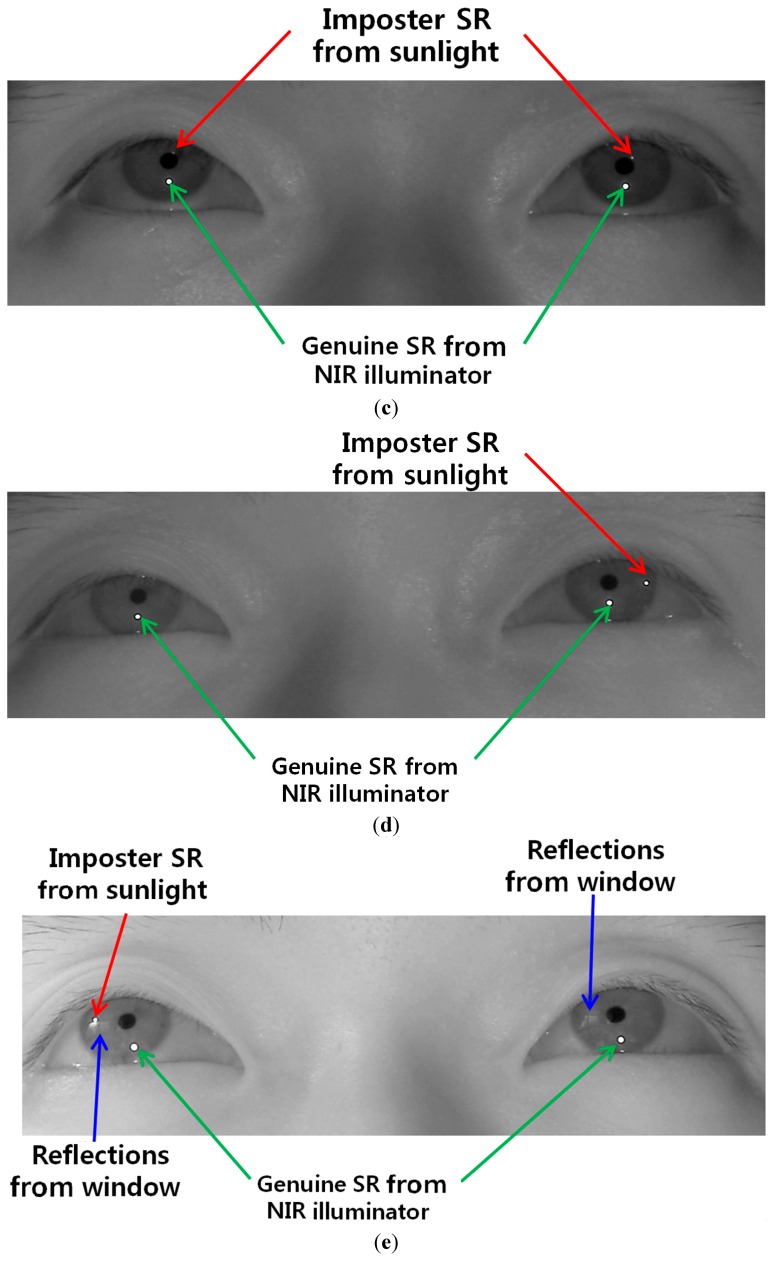

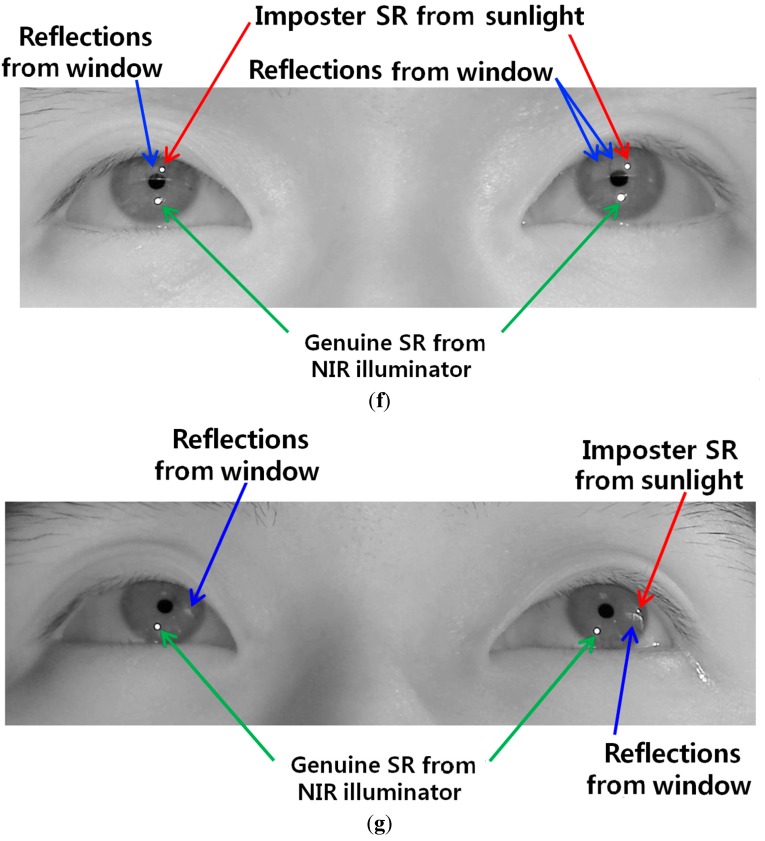
Comparative experiments in various positions of sunlight relative to user’s face. (**a**) the left, middle, and right images show the cases when sunlight is positioned in the left side, front, and right side of user’s face, respectively; (**b**) the captured eye image of the case of left image of (a) with BPF; (**c**) the captured eye image of the case of middle image of (a) with BPF; (**d**) the captured eye image of the case of right image of (a) with BPF; (**e**) the captured eye image of the case of left image of (a) with LPF; (**f**) the captured eye image of the case of middle image of (a) with LPF; (**g**) the captured eye image of the case of right image of (a) with LPF.

We performed additional experiments where the presence of reflections in the user’s iris can occur due to window frames or glasses. As shown in Figure 27a, three experiments were done. The first case is when sunlight is positioned in the left side of user’s face as shown in the left image of Figure 27a. The second case is when the sunlight is positioned in front of user’s face as shown in the middle image of Figure 27a. The third one is when the sunlight is positioned in the right side of user’s face as shown in the right image of Figure 27a.

Figure 27b–d represents the captured eye images of the cases of the left, middle, and right images of Figure 27a with BPF, respectively. As shown in Figure 27b–d, we can find that there is no reflection caused by the window frames or glasses in the user’s iris. This is because our system uses a 850 nm NIR light illuminator and the camera attached by a BPF (passing range of 850 ± 25 nm) [20,21]. Therefore, most of transmitted light to the camera of our system is that of NIR illuminator (850 nm) of our gaze detection system, and other light of different wavelengths in addition to the reflections caused by window frames or glasses can be blocked out by the BPF of our system.

For comparison, we show the eye images captured with LPF. Figure 27e–g represents the captured eye images of the cases of the left, middle, and right images of Figure 27a with LPF, respectively. As shown in Figure 27e–g, we can find that there exist reflections produced by the window frames or glasses in the user’s iris when the LPF which passes the NIR light whose wavelength is same as (longer than) 700 nm is used in our gaze detection system. From these, we can find that the reflections produced by window frames or glasses can be blocked out by the BPF of our system. We include the experimental results of the percentage of correctly labeled image of genuine SR *versus* total number of image in the four cases (Figure 25a–d) as shown in Table 2.

**Table 2:** The percentage of correctly labeled image of genuine SR *versus* total number of image in the four cases (Figure 25a–d, unit: %).

Case	Percentage
Figure 25a	100
Figure 25b	90.74
Figure 25c	100
Figure 25d	90.37
Average	95.28

As shown in Table 2, the average percentage of correctly labeled images of genuine SR *versus* total number of images is higher than 95%. In Table 2, the percentages in the cases of Figure 25b,d are lower than those of Figure 25a,c. That is because these cases of Figure 25b,d correspond to the third scenario of Figure 16, and the errors occur when the pupil center belongs to the common area of GPMA and IPMA.

**Figure 28:**
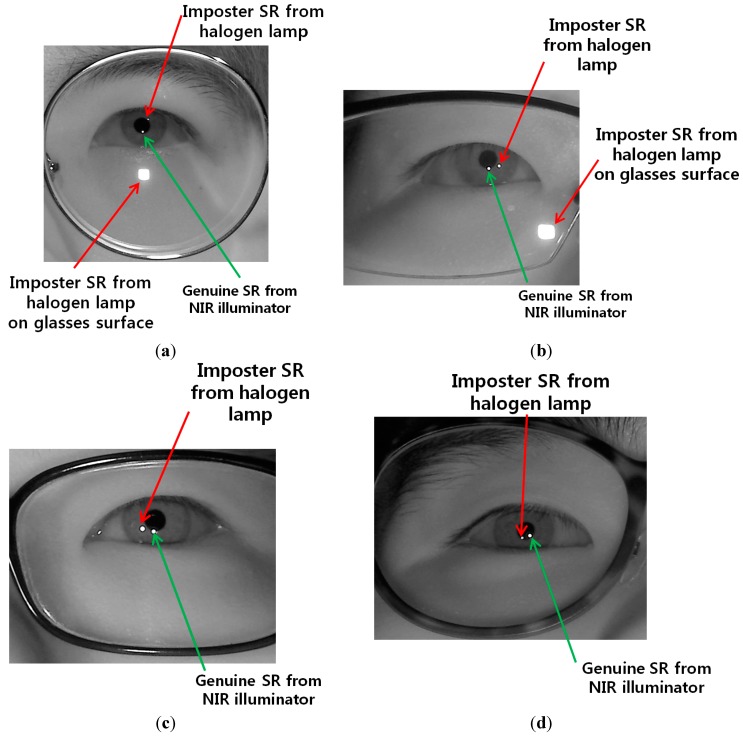
Examples of users wearing glasses. (**a**) user 1 with the halogen lamp above monitor display; (**b**) user 2 with the halogen lamp at the right of monitor display; (**c**) user 3 with the halogen lamp at the left of monitor display; (**d**) user 4 with the halogen lamp below monitor display.

**Figure 29:**
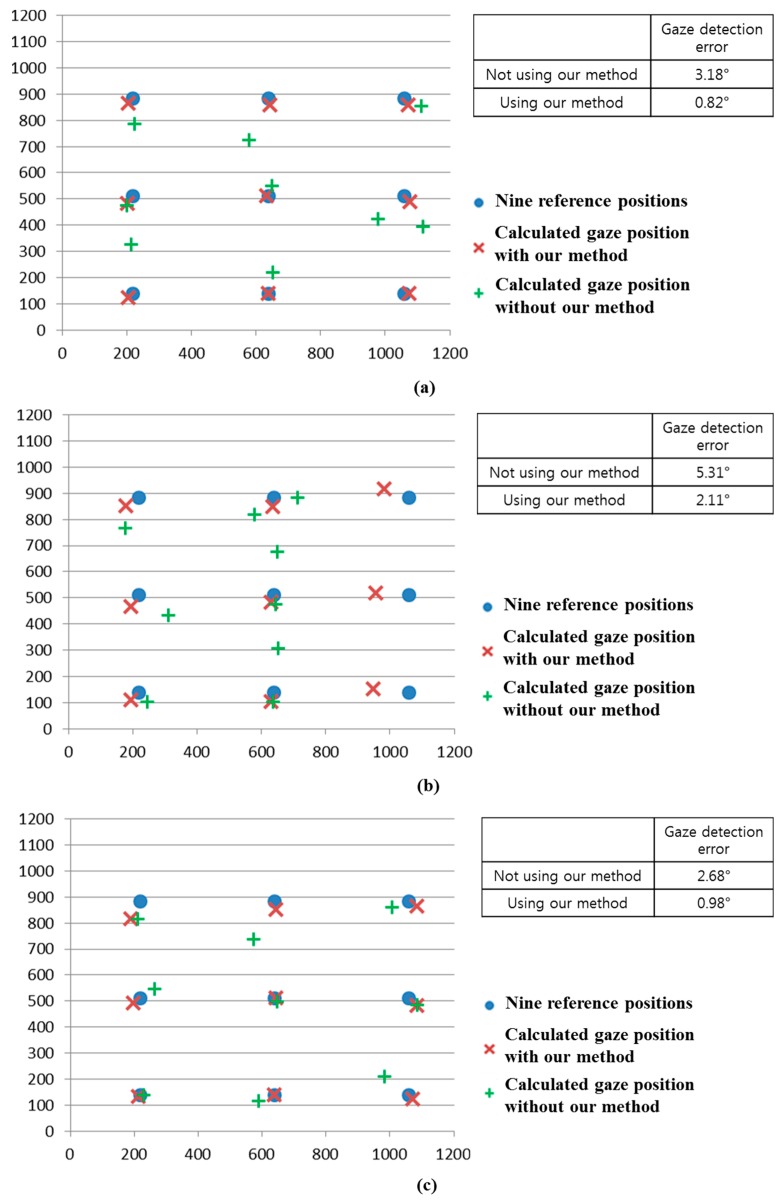

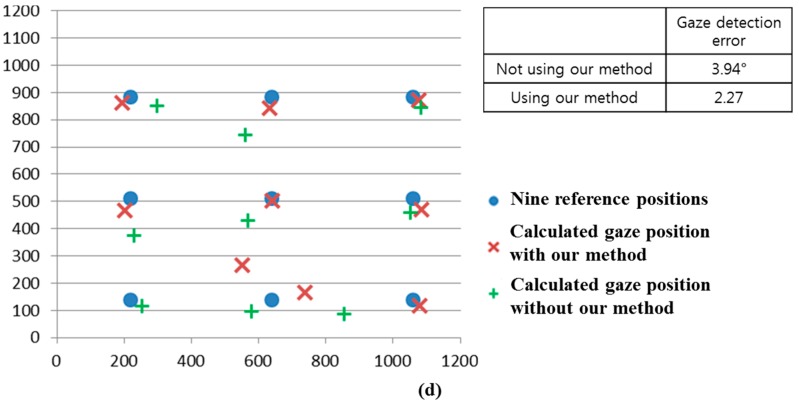
Comparisons of gaze detection errors with five people wearing glasses without and with our method of Section 2.5 in the presence of the halogen lamp at various positions (like Figure 25) (**a**) above monitor display; (**b**) right of monitor display; (**c**) below monitor display; (**d**) left of monitor display.

As the next experiment, we performed additional experiments with five people wearing glasses. Figure 28 shows the examples of users wearing glasses. Results are shown in Figure 29 and Table 3. Like the previous experiments of Figure 24 and Figure 26, we measured the gaze detection error when each person gazes at the nine reference points of Figure 29. In these cases, each calculated gaze position is obtained by averaging all the gaze positions calculated from five people. As shown in Figure 29, the gaze detection errors with people wearing glasses were almost similar to those with people not wearing glasses of Figure 26.

In addition, we include the experimental results of the percentage of correctly labeled images of genuine SR *versus* total number of image in the four cases (Figure 29a–d) as shown in Table 3, where the average percentage of correctly labeled image of genuine SR *versus* total number of images is higher than 96%, which is similar to that with people without glasses of Table 2. From Figure 29 and Table 3, we can find that the performance of our system is not affected by people wearing glasses.

**Table 3:** The percentage of correctly labeled image of genuine SR *versus* total number of image in case of five people wearing glasses (unit: %).

Case	Percentage
Figure 29a	100
Figure 29b	93.33
Figure 29c	100
Figure 29d	91.11
Average	96.11

Although the gaze detection error with our method is much lower than that without our method in Figure 29a–d, the errors with our method in case of Figure 29b,d are higher than those of Figure 29a,c. In addition, the percentages of correctly labeled image of genuine SR *versus* total number of image in case of Figure 29b,d are lower than those of Figure 29a,c in Table 3. That is because these cases of Figure 29b,d correspond to the third scenario of Figure 16, and the errors occur when the pupil center belongs to the common area of GPMA and IPMA.

Because it is difficult to obtain the images with sunlight in various positions, we have experimented with halogen illuminators placed in various positions as shown in Figure 25. Our system uses an 850 nm NIR light illuminator and the camera is equipped with a BPF (passing range of 850 ± 25 nm) [20,21], which makes the wavelength of most of the light transmitted to the camera of our system be within the range around 850 nm. In addition, when we compared the power of the halogen illuminators and sunlight in the wavelength range around 850 nm, they are similar. From that, we can conclude that the effect of sunlight is expected to be similar to that of the halogen illuminators.

**Figure 30:**
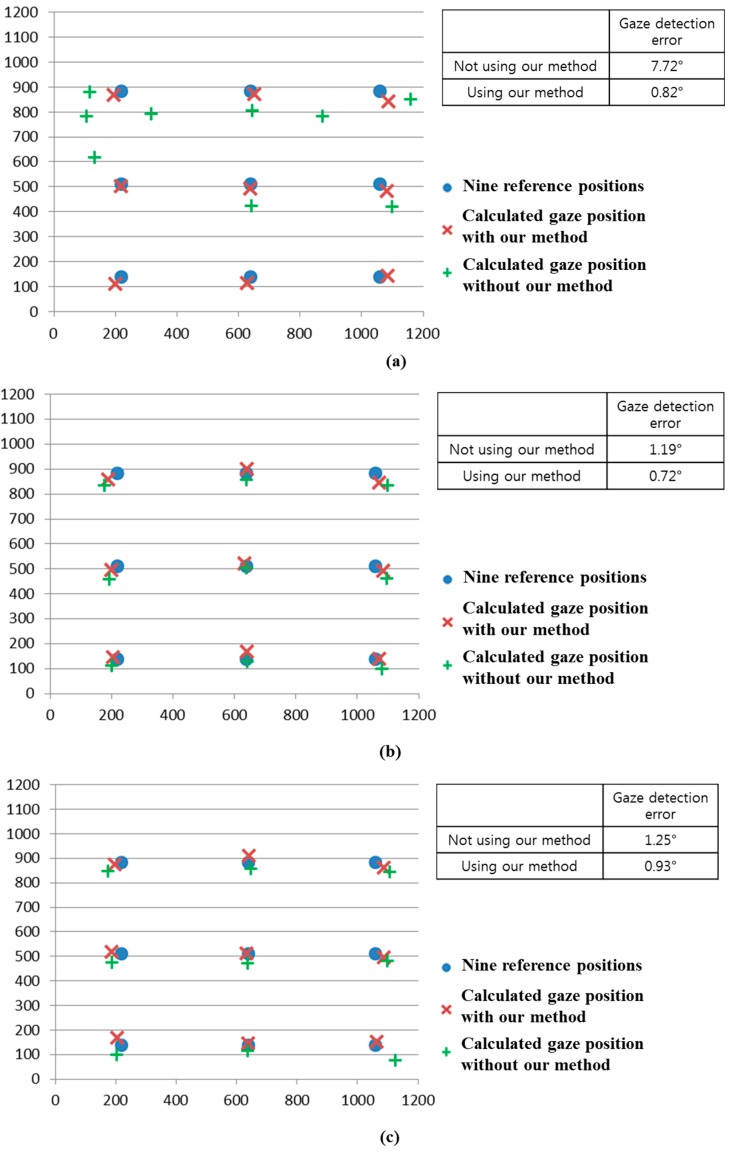
Comparisons of gaze detection errors without and with our method of Section 2.5 in various positions of sunlight relative to user’s face (**a**) when sunlight is positioned in front of user’s face; (**b**) when sunlight is positioned in the right side of user’s face; (**c**) when sunlight is positioned in the left side of user’s face.

To prove this, we performed additional experiments using various sunlight positions relative to the user’s face (as shown in Figure 27). As shown in Figure 30 and Table 4, the results (gaze detection error and the percentage of correctly labeled image of genuine SR *versus* total number of image) from five people are similar to those of Figure 25 and Table 2. From that, we can find that the performance of our system is not affected by the various positions of sunlight.

**Figure 31:**
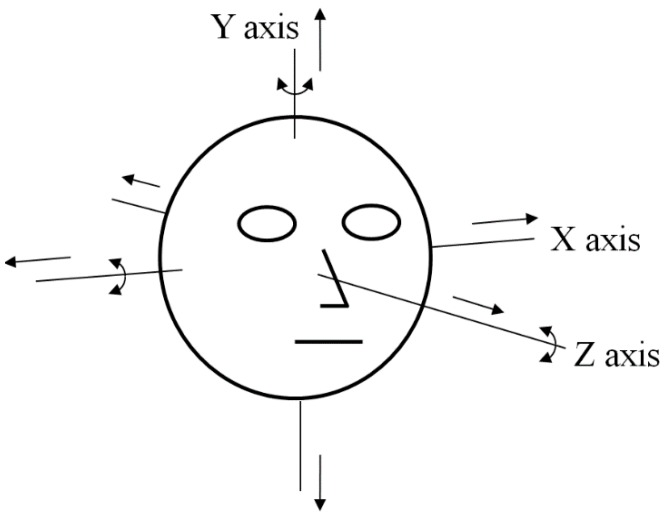
Head rotation and translation in our experiments.

**Figure 32:**
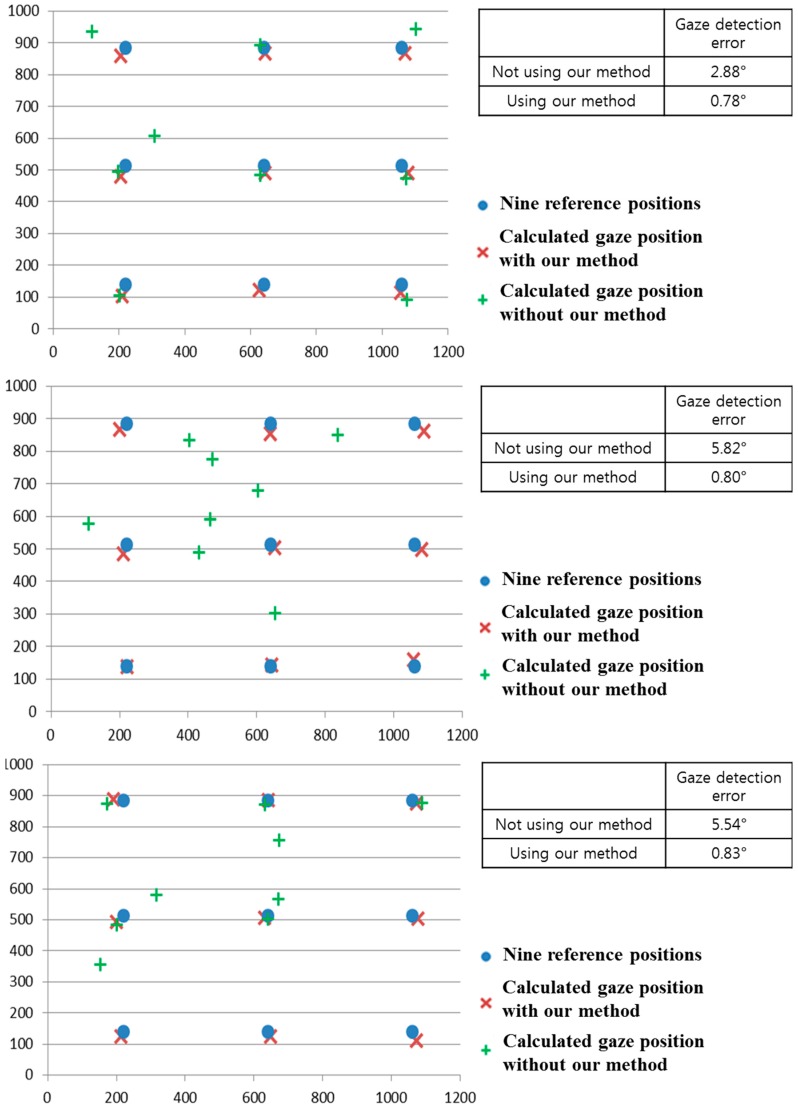

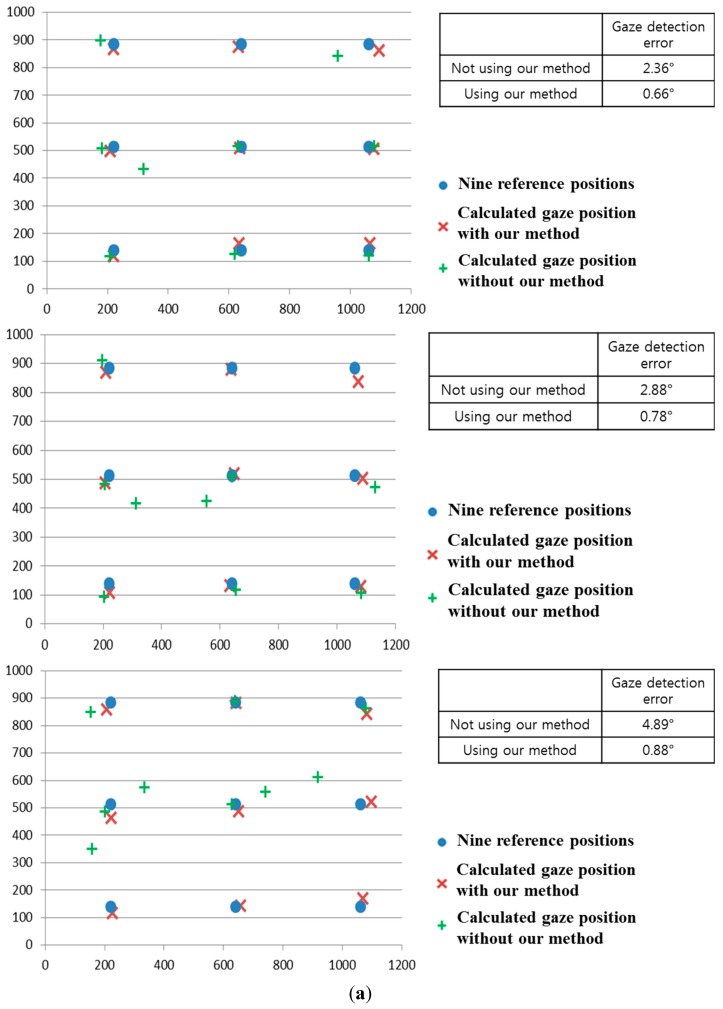

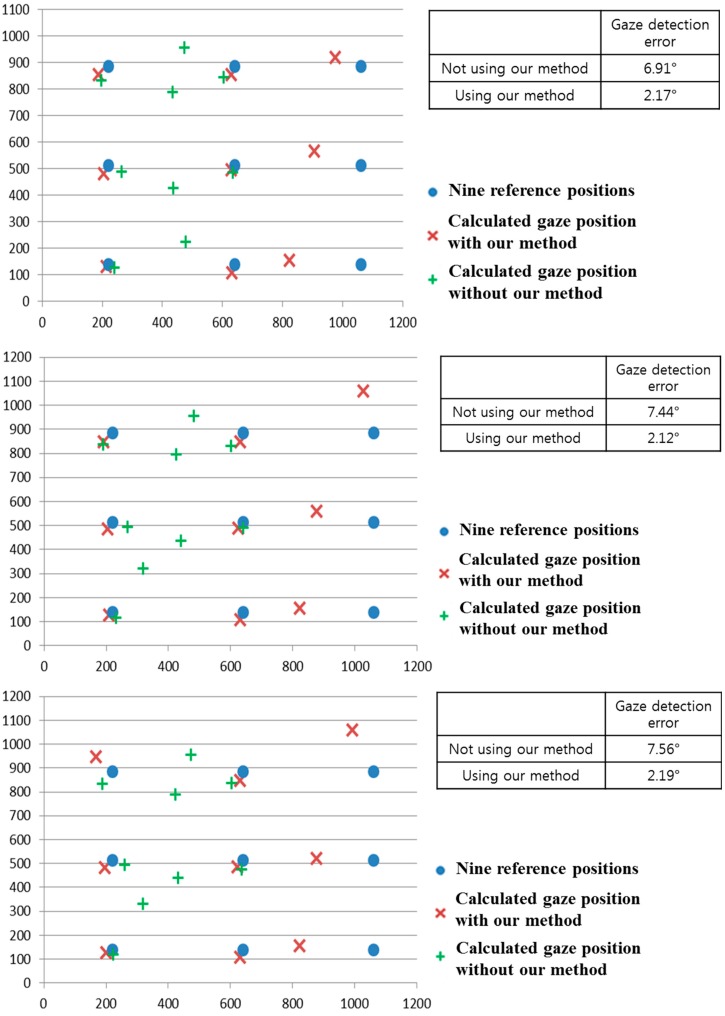

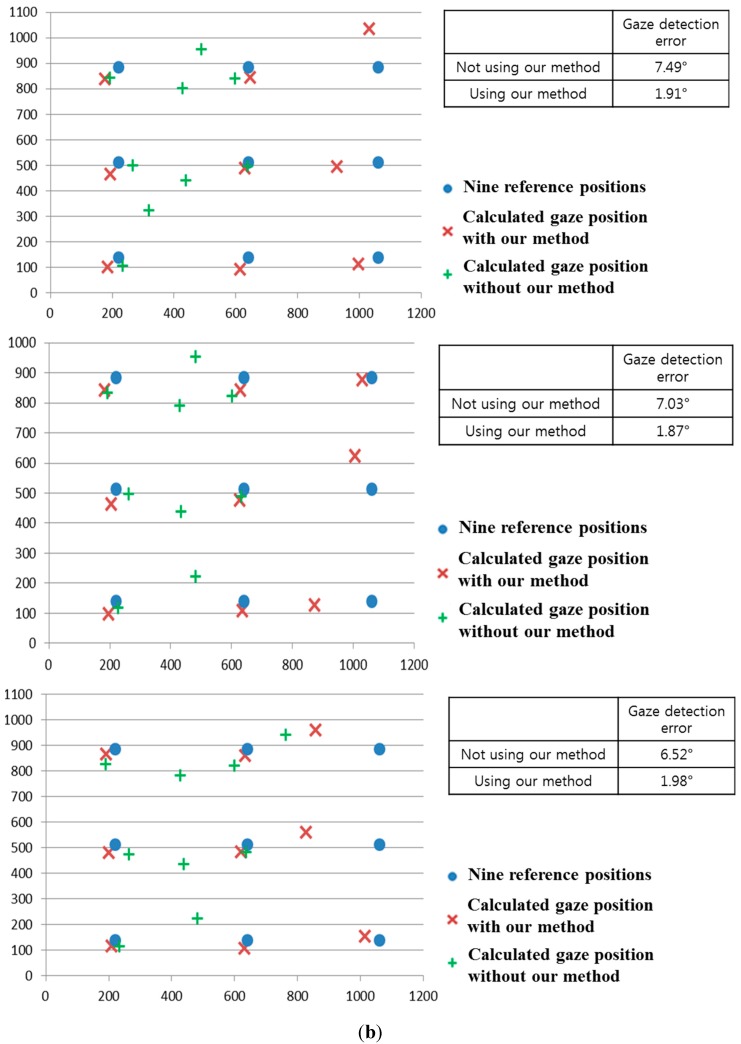

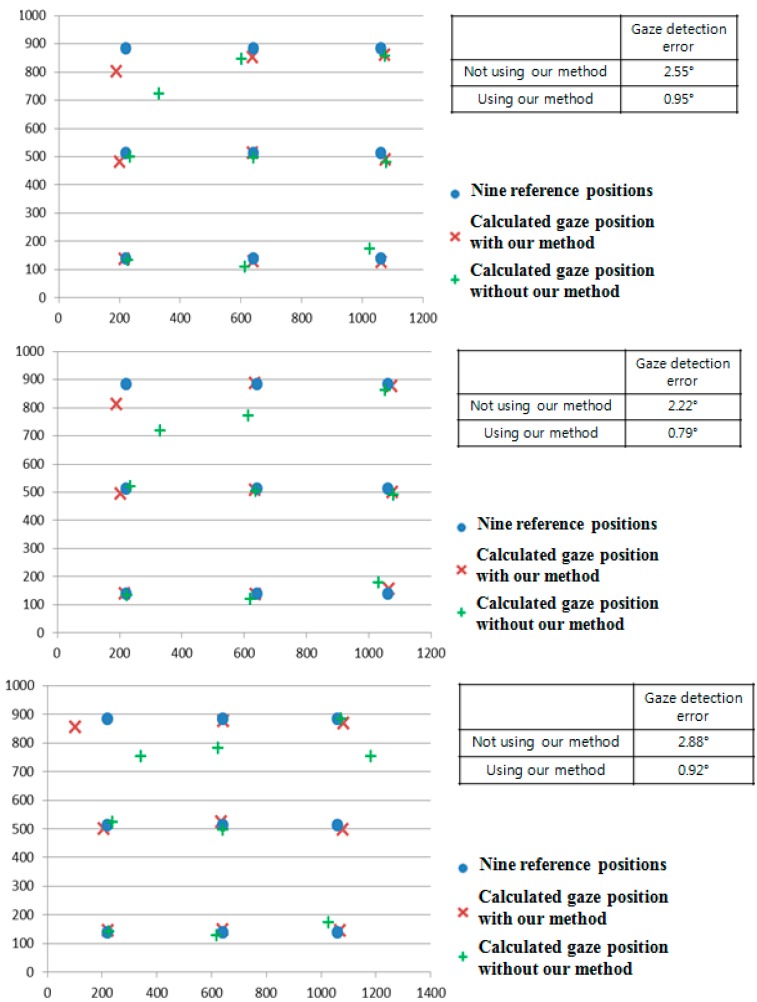

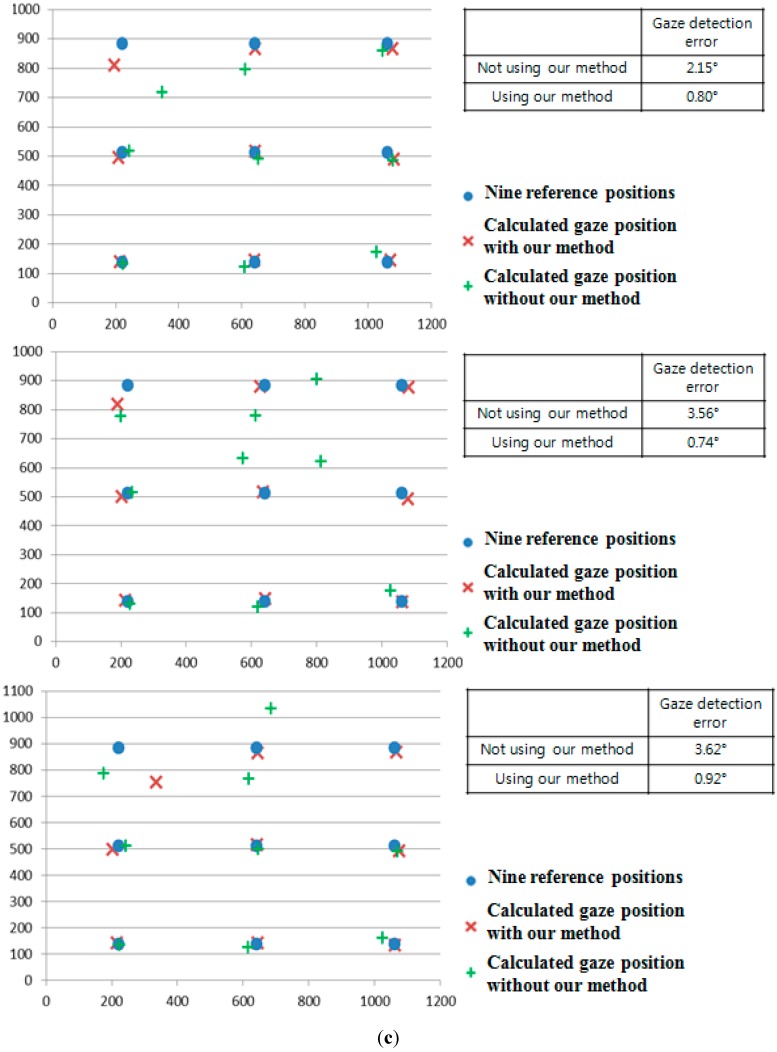

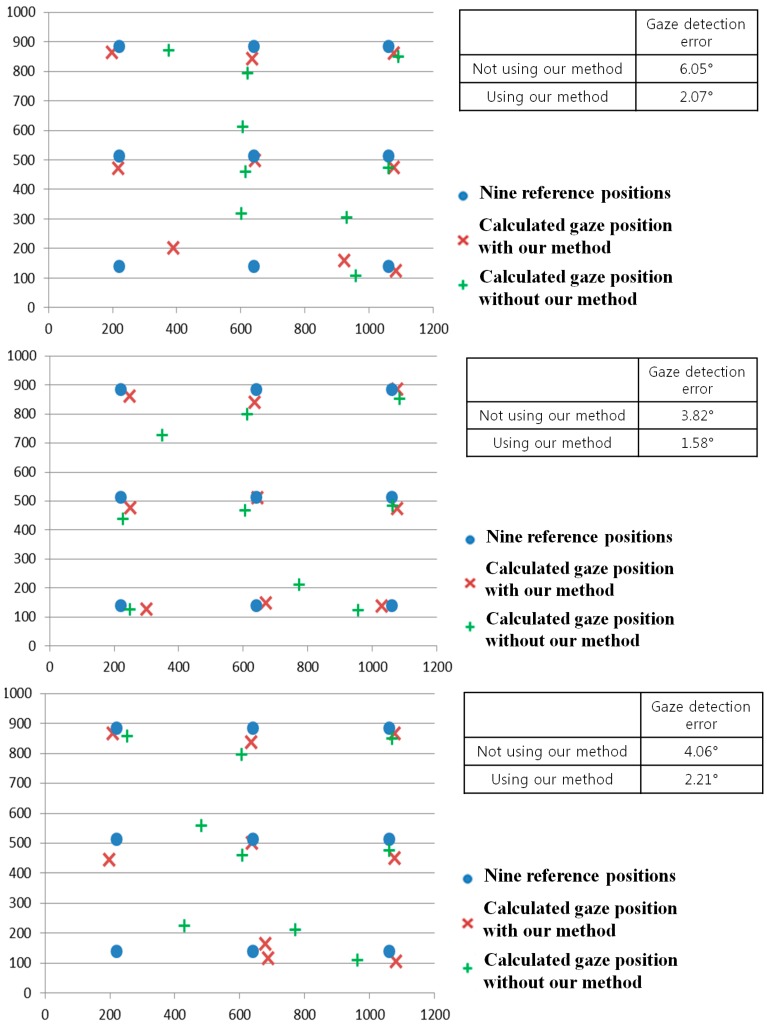

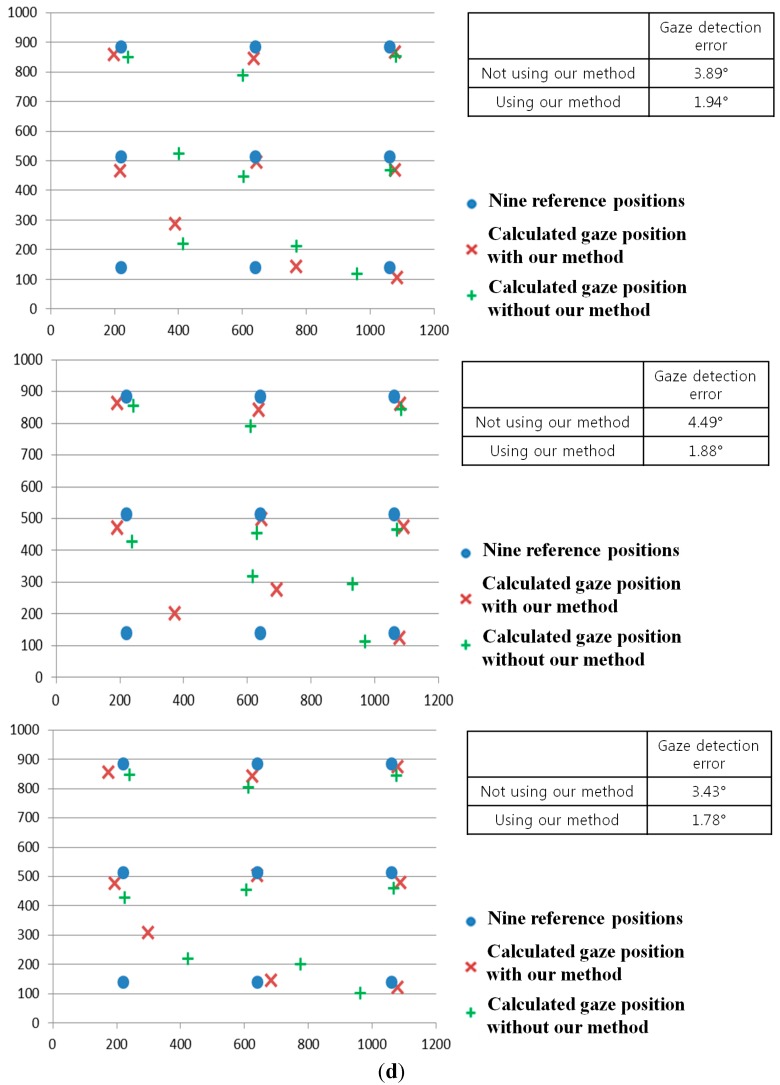
Comparisons of gaze detection errors with five people having various head rotations and translations without and with our method of Section 2.5 in the presence of the halogen lamp at various positions (like Figure 25). (**a**) above monitor display; (**b**) right of monitor display; (**c**) below monitor display; (**d**) left of monitor display (In the Figure 29a–d, the 1st–6th Figures represent the cases of head rotations in X, Y, Z axes, and head translations in X, Y, Z axes of Figure 31, respectively).

As the next experiment, we performed additional tests with five people performing various head rotations and translations as shown in Figure 31. Like the previous experiments shown in Figure 24, Figure 26, Figure 29 and Figure 30, we measured the gaze detection error when each person gazed at nine reference points (Figure 32). In these cases, each calculated gaze position is obtained by averaging all the gaze positions calculated from the five people. In detail, each people naturally rotated his head five times based on the X, Y, and Z axes, respectively, within an angular range of about −10°~+10°. This angular range is determined considering the case when a user naturally looks at any position of the monitor with head rotation. In addition, each person naturally translated his head five times in the directions of X, Y, and Z axes, respectively, within the translational range of about –4~+4 cm. This translational range is also determined considering the case when a user naturally looks at any position of the monitor with head translation. 

**Table 4:** The percentage of correctly labeled image of genuine SR *versus* total number of image in case of sunlight (unit: %).

Case	Percentage
Figure 30a	100
Figure 30b	100
Figure 30c	100
Average	100

In each case, we performed the experiments with the halogen lamp in four positions (above, right, below, and left of monitor display) while each people looked at the nine reference positions to measure the accuracy of gaze detection as shown in Figure 32. Therefore, we obtained the 5400 images of (5 people × 6 head movements (3 rotations and 3 translations) × 4 positions of halogen lamp × 9 images (image where a user gazed at one of the nine reference positions of monitor) × 5 trials) for measuring the accuracies.

As shown in Figure 32, the gaze detection errors with people performing various head rotations and translations were almost similar to those with people not performing head rotations and translations (Figure 26). 

In addition, we include the experimental results of the percentage of correctly labeled images of genuine SR *versus* total number of image in the twenty four cases (Figure 32a–d) as shown in Table 5. As shown in Table 5, the average percentage of correctly labeled image of genuine SR *versus* total number of image is higher than 95%, which is similar to that with people not having head rotations and translations of Table 2. From the Figure 32 and Table 5, we can find that the performance of our system is not affected by head rotations or translations.

**Table 5:** The percentage of correctly labeled image of genuine SR *versus* total number of image in case of head rotations and translations (unit: %).

Case		Percentage
Figure 32a	1st Figure	100
	2nd Figure	100
	3rd Figure	100
	4th Figure	100
	5th Figure	100
	6th Figure	100
Figure 32b	1st Figure	91.11
	2nd Figure	91.11
	3rd Figure	93.33
	4th Figure	91.11
	5th Figure	91.11
	6th Figure	91.11
Figure 32c	1st Figure	100
	2nd Figure	100
	3rd Figure	100
	4th Figure	100
	5th Figure	100
	6th Figure	100
Figure 32d	1st Figure	91.11
	2nd Figure	93.33
	3rd Figure	91.11
	4th Figure	91.11
	5th Figure	91.11
	6th Figure	91.11
	Average	95.74

As shown in Figure 14, Figure 15 and Figure 16 and Equations (1) and (2), discrimination between the correct SR and the imposter SR was possible by verifying the relationship between the corneal SR and the pupil movable area with the relative position of the pupil and the corneal SR.

**Figure 33:**
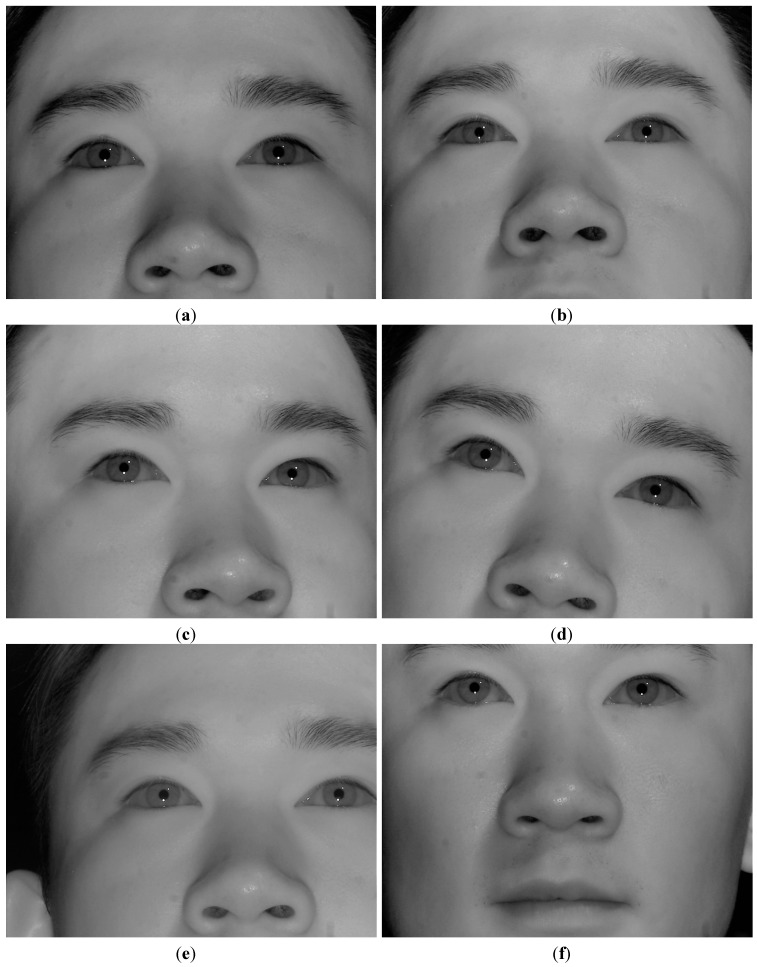

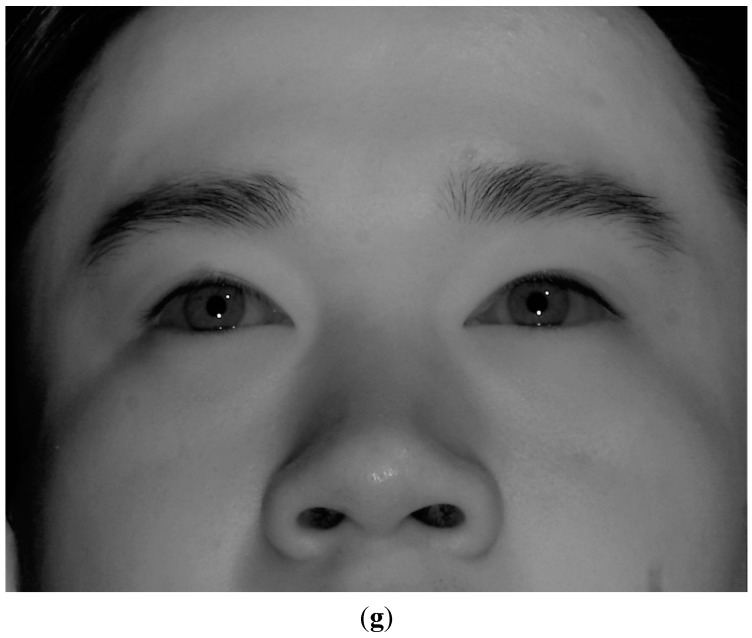
The examples of relationships among GPMA, IPMA, correct SR, and imposter SR in case of head rotations and translations while gazing at same position. The directions of rotations and translations are shown in Figure 31. In each eye image of Figure of (**a**)~(**g**), upper and lower SRs are imposter and correct SRs, respectively. (**a**) before head movement; (**b**) head rotation in X axis; (**c**) head rotation in Y axis; (**d**) head rotation in Z axis; (**e**) head translation in X axis; (**f**) head translation in Y axis; (**g**) head translation in Z axis.

In the cases of head rotations and translations, the direction and amount of movement of the correct SR are almost same to those of the imposter SR in the image, as shown in Figure 33. Therefore, the relative positions among the correct SR, the imposter SR, and pupil center are maintained in case of gazing at same position even with head rotations and translations as shown in Figure 33. Consequently, the relationships among GPMA, IPMA, correct SR, and imposter SR (Figure 14, Figure 15 and Figure 16) are almost maintained before and after head movement as shown in Figure 33. Therefore, the performance of our system is not affected by head rotations and translations as shown in Figure 32 and Table 5.

In previous research, the adaptive boosting (AdaBoost) detector has been widely used for eye ROI detection [24]. However, the AdaBoost detector can only locate the rough position of eye ROI (like Step 3 of Figure 1). Therefore, the accurate pupil center and corneal SR center should be detected by our method (explained in Section 2.3) in order to calculate the gaze position. As the next experiment, we compared the performances of eye ROI detection by the AdaBoost detector, another statistical continuously adaptive mean shift (Camshift) method [25], and our method with the data of Figure 25, Figure 29 and Figure 30. As shown in Table 6, the accuracies of eye ROI detection by AdaBoost or the Camshift detector are lower than that by our method. In addition, the processing time of eye ROI detection by AdaBoost or Camshift detector is higher than that of our method (17 ms). From that, we can conclude that our method outperforms these other methods.

**Table 6:** Comparisons of the accuracies and processing time of eye ROI detection by AdaBoost, Camshift, and our method.

Method	Accuracy (%)	Processing Time (ms)
AdaBoost method	96.25	104.985
Camshift method	99.5	45.8675
Our method	100	17

As the last experiment, we measured the processing time of our method (according to the steps of Figure 1) as shown in Table 7. From the experimental results, we can know that our system can be operated at the fast speed of approximately 19 (1000/53) frames/sec.

**Table 7:** Processing time of our method (unit: ms).

Steps	Processing Time
Detect the largest area by external light	1
Detect the eye ROI	16
Find the threshold value for binarization of pupil	16
Detect SR and pupil	20
Discriminate genuine SR from imposter one by external light	0
Calculate gaze position	0
Total	53

## 4. Conclusions

In this research we propose a new gaze detection system that is robust to the effects of external light. BPF was found to be more effective at reducing the influence of external light than LPF. By using component labeling and size filtering, the erroneous regions of external light in the captured images were removed, even in cases where the external light is positioned behind the user. The discrimination between the correct and imposter SRs was possible. This was done by verifying the relationship between the corneal SR and the pupil movable area with the relative position of the pupil and the corneal SR. In addition, a new pupil detection method is proposed based on the histogram of the eye region which is processed by an average filter of 5×5 pixels. Through experiments where a halogen lamp is placed at various positions, we can confirm that our gaze detection system is robust to the external light effects.

In future work, we will research the method to reduce the gaze detection errors by solving the problem of the third scenario (the case where the pupil center belongs to the common area of GPMA and IPMA) by using tracking information in successive frames. In addition, we will test our system in more various environments such as outdoors, mobile and actual car environments. These are expected to make our system more robust to external light.
